# Identification of flavonoids from natural products against hyperlipidaemia through computational approaches

**DOI:** 10.1371/journal.pone.0346073

**Published:** 2026-10-09

**Authors:** Nurnazira Hanis Mustaffar Kamal, Nurul Azira Ismail, Ernie Zuraida Ali

**Affiliations:** 1 School of Graduate Studies, Postgraduate Centre Management and Science University, University Drive, Shah Alam, Selangor, Malaysia; 2 Faculty of Health and Life Sciences, Management and Science University, University Drive, Shah Alam, Selangor, Malaysia; 3 Institute for Medical Research, National Institutes of Health Malaysia, Shah Alam, Selangor, Malaysia; Universidade Federal do Para, BRAZIL

## Abstract

Over the centuries, hyperlipidaemia has been recognised as a major risk factor contributing to cardiovascular disease (CVD). Current research increasingly explores natural compounds such as flavonoids for their potential lipid-lowering effects. This study evaluated pharmacological potential of flavonoids to identify strong inhibitors for target receptor, Proprotein Convertase Subtilisin/Kexin Type (PCSK9). A total of 29 compounds, including one FDA-approved drugs were screened for drug-likeness and ADMET properties, followed by molecular docking and Molecular Dynamics (MD) simulations using PCSK9 receptor containing glucose and sodium (PDB ID: 5OCA). This complex was chosen due to their functional relevance to cholesterol metabolism. As a results, 24 compounds satisfied Lipinski’s Rule of Five and exhibited favourable drug-likeness and toxicity profiles. Simvastatin (control) was discovered to bind into the pocket B binding site along with seven flavonoids; Diosmetin, Eupatorin, Isorhamnetin, Myricetin, Petunidin, Sinensetin, and Tilianin. These flavonoids displayed docking energies ranging from −8.0 to −10.2 kcal/mol. Tilianin showed the strongest affinity (−10.2 kcal/mol), while Simvastatin bound with −9.1 kcal/mol. MD simulations confirmed that the PCSK9-ligand complexes remained structurally stable throughout a 100 ns trajectory. MM/PBSA analysis further supported the binding interactions, revealing free energies values ranging from −17 to −30.41 kcal/mol. Among the flavonoids evaluated, Tilianin, Myricetin, and Sinensetin demonstrate the most favourable interaction profiles. These finding suggest that the selected flavonoids have strong potential as promising anti-hyperlipidaemic candidates targeting PCSK9.

## 1. Introduction

Cardiovascular disease (CVD) remains the leading cause of mortality worldwide and continues to pose a major public health challenge. CVD encompasses a group of disorders affecting the heart and blood vessels, including coronary artery disease, cerebrovascular disease, and peripheral arterial disease. According to the American Heart Association, approximately 19.1 million deaths in 2020 were attributed to CVD, making it the leading cause of death globally [1]. Among the modifiable risk factors, elevated blood cholesterol is one of the strongest contributors to the development and progression of CVD, particularly through the acceleration of atherosclerosis [2]. In Malaysia, hypercholesterolemia affects approximately 47.7% of adults, with 38.6% of cases remaining undiagnosed, highlighting the urgent need for effective and accessible therapeutic strategies [3].

Lipoproteins are essential macromolecular complexes responsible for transporting cholesterol, triglycerides, and other lipids throughout the circulatory system. Since lipids are water-insoluble, lipoproteins facilitate their transport to peripheral tissues for energy production, membrane synthesis, and hormone biosynthesis [4,5]. Based on their density and composition, lipoproteins are classified into high-density lipoprotein (HDL), low-density lipoprotein (LDL), and very-low-density lipoprotein (VLDL) [6]. VLDL transports triglycerides synthesized in the liver and is subsequently metabolized into LDL, the primary carrier of circulating cholesterol. Elevated LDL-cholesterol (LDL-C) levels promote cholesterol accumulation within arterial walls, leading to atherosclerotic plaque formation and an increased risk of cardiovascular events, whereas HDL facilitates reverse cholesterol transport and exerts a protective effect against CVD [7].

Proprotein convertase subtilisin/kexin type 9 (PCSK9) has emerged as a critical regulator of cholesterol homeostasis and represents the third major genetic cause of familial hypercholesterolemia [8]. The human PCSK9 gene, located on chromosome 1q32.3, consists of 13 exons encoding a 692-amino acid protein [9,10]. PCSK9 expression is primarily regulated by sterol regulatory element-binding protein-2 (SREBP-2), a master transcription factor involved in cholesterol metabolism, while hepatocyte nuclear factor-1 alpha (HNF-1α) further enhances its transcriptional activity [11]. Secreted PCSK9 binds to the epidermal growth factor-like repeat A (EGF-A) domain of the low-density lipoprotein receptor (LDLR), directing the receptor toward lysosomal degradation rather than recycling to the hepatocyte surface [12]. Consequently, hepatic LDLR abundance is reduced, limiting LDL-C clearance from the circulation and increasing plasma cholesterol concentrations [13]. Gain-of-function mutations in PCSK9 are associated with severe hypercholesterolemia and increased cardiovascular risk, whereas loss-of-function variants significantly reduce circulating LDL-C levels and protect against CVD [14]. These findings have established PCSK9 as an attractive therapeutic target for the treatment of hypercholesterolemia.

Current lipid-lowering therapies, including statins and PCSK9 monoclonal antibodies, have significantly improved the management of hypercholesterolemia. Statins remain the first-line treatment by inhibiting 3-hydroxy-3-methylglutaryl coenzyme A (HMG-CoA) reductase and reducing endogenous cholesterol synthesis. However, statin intolerance, adverse effects, and variable patient responses continue to limit long-term treatment adherence. Although monoclonal antibodies targeting PCSK9 effectively reduce LDL-C levels, their high cost, injectable route of administration, and limited accessibility restrict their widespread clinical application, particularly in low- and middle-income countries [15]. These limitations have stimulated increasing interest in identifying orally active, cost-effective small-molecule inhibitors capable of modulating PCSK9 function.

Natural products have historically served as valuable sources for drug discovery, with flavonoids representing one of the largest classes of bioactive polyphenolic compounds. Increasing evidence indicates that flavonoids exert lipid-lowering effects by regulating cholesterol metabolism. Several flavonoid subclasses suppress SREBP-2 and HNF-1α, leading to reduced PCSK9 expression, enhanced LDLR expression, and improved hepatic LDL-C clearance [16]. Beyond these transcriptional effects, the polyphenolic scaffold of flavonoids, characterized by aromatic rings and multiple hydroxyl groups, enables the formation of hydrogen bonds, hydrophobic interactions, and π-interactions with proteins [17]. These structural features suggest that flavonoids may also directly interact with PCSK9 [18], although this mechanism remains largely unexplored. In addition, flavonoids have been shown to inhibit cholesterol biosynthesis, attenuate oxidative stress, reduce vascular inflammation, and improve endothelial function, collectively contributing to cardiovascular protection [19]. For example, Talinum triangulare leaf flavonoid extract has been reported to ameliorate dyslipidemia primarily through inhibition of HMG-CoA reductase, thereby reducing hepatic cholesterol synthesis. Additionally, the extract improves hyperglycemia by enhancing plasma insulin secretion, inhibiting α-amylase activity, and reducing oxidative stress [20]. Likewise, flavonoids such as quercetin, rutin, luteolin derivatives, and other naturally occurring compounds have demonstrated beneficial effects against dyslipidemia and atherosclerosis by improving lipid homeostasis and reducing inflammatory responses [21–23].

Recent advances in computer-aided drug discovery (CADD), including molecular docking, molecular dynamics (MD) simulations, and binding free-energy calculations, have accelerated the identification of potential therapeutic compounds from natural products while reducing the time and cost associated with conventional experimental screening [24]. These computational approaches enable detailed characterization of protein-ligand interactions, conformational stability, binding energetics, and pharmacokinetic properties, thereby providing valuable mechanistic insights and facilitating the prioritization of promising drug candidates before experimental validation. Recent studies have successfully applied integrated computational workflows to identify bioactive compounds against diverse therapeutic targets, demonstrating the reliability and increasing importance of these approaches in modern drug discovery [25–27].

Despite the growing evidence supporting the lipid-lowering properties of flavonoids, relatively few studies have systematically investigated their direct interactions with PCSK9 at the molecular level. Most published studies [28] have focused on gene expression or serum lipid profiles, whereas comprehensive computational investigations of flavonoid-PCSK9 interactions remain limited. Furthermore, the structural mechanisms underlying flavonoid-mediated modulation of PCSK9 have yet to be fully elucidated. Therefore, this study aimed to systematically identify naturally occurring flavonoids with favorable drug-like properties and stable binding interactions with PCSK9 using an integrated computational workflow. The findings may facilitate the discovery of promising flavonoid-based lead compounds for the future development of affordable and effective anti-hyperlipidemic therapies targeting PCSK9.

## 2. Materials and methods

This study employed a four-stage computational approach involving ligand preparation and toxicity screening, protein structure refinement and validation, molecular docking, molecular dynamics (MD) simulation and MM/PBSA binding free-energy analysis. A systematic workflow was implemented to identify potential PCSK9 inhibitors as illustrated in Fig 1.

**Fig 1:**
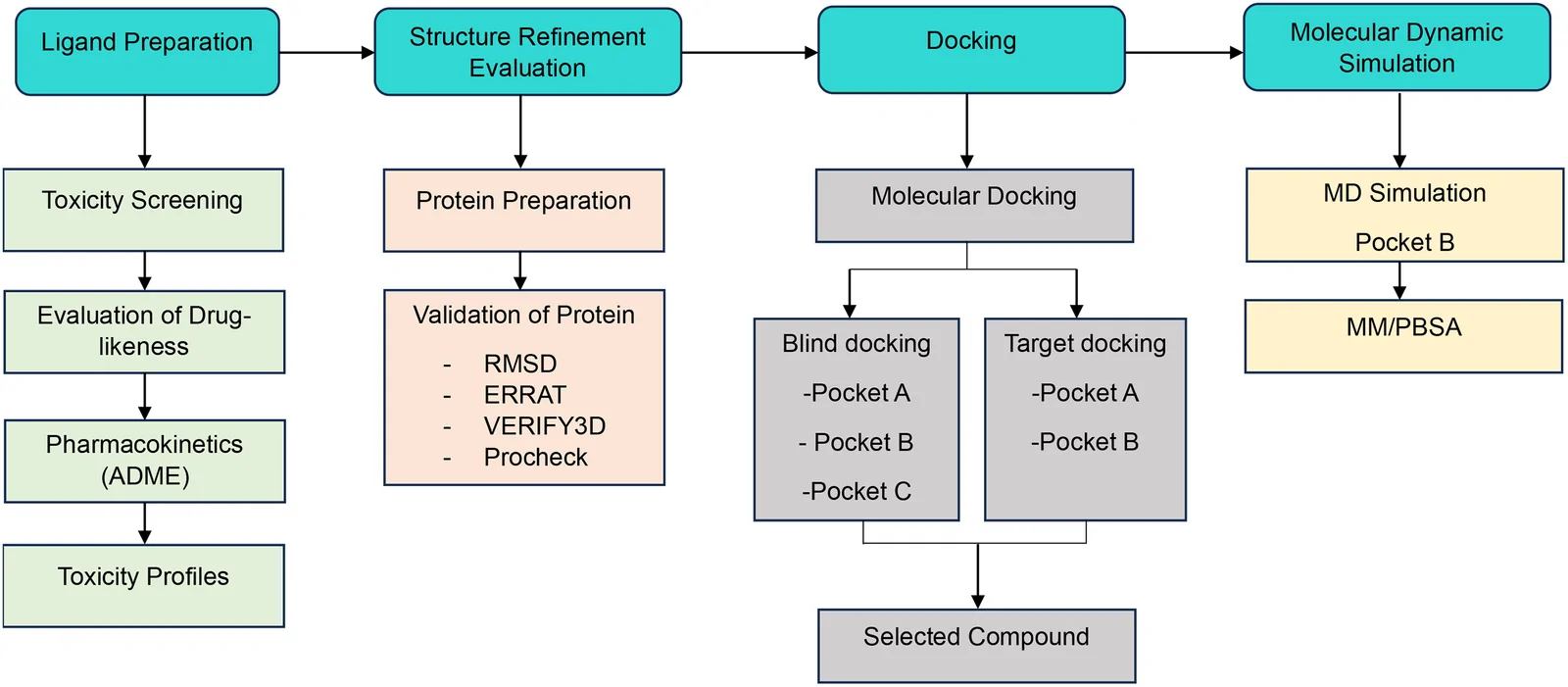
Overview of the methodological framework applied in this research. The workflow comprises ligand preparation, drug-likeness and ADME evaluation, toxicity screening, protein preparation and validation, molecular docking, molecular dynamics simulation, and MM/PBSA analysis.

### 2.1. Ligand preparation and toxicity screening

#### 2.1.1. Ligand preparation.

Twenty-nine compounds comprising twenty-eight naturally occurring flavonoids and one FDA-approved drug (Simvastatin) used as the reference control were selected based on an extensive literature review (Table 1). Two-dimensional (2D) chemical structures were retrieved from the PubChem database (https://pubchem.ncbi.nlm.nih.gov/) in SDF format. The structures were converted into three-dimensional (3D) conformations using Open Babel version 3.1.1 [29].

**Table 1:** Flavonoids and Pubchem CID used in this study.

	Flavonoid	Pubchem CID		Flavonoid	Pubchem CID
1)	Apigenin	5280443	16)	Isorhamnetin	5281654
2)	Apigenin-7-O-neohesperidoside	5282150	17)	Kaempferol	5280863
3)	Baicalein	5281605	18)	Luteolin	5280445
4)	Baicalin	64982	19)	Myricetin	5281672
5)	Catechin	9064	20)	Naringenin	439246
6)	Cyanidin	128861	21)	Petunidin	441774
7)	Daidzein	5281708	22)	Pinocembrin	68071
8)	Delphinidin	68245	23)	Quercetin	5280343
9)	Diosmetin	5281612	24)	Rutin	5280805
10)	Epiafzelechin	443639	25)	Simvastatin (Control)	54454
11)	Eupatorin	97214	26)	Sinensetin	145659
12)	Fisetin	5281614	27)	Taxifolin	439533
13)	Formononetin	5280378	28)	Theaflavin	135403798
14)	Gallocatechin	65084	29)	Tilianin	5321954
15)	Hesperidin	10621			

#### 2.1.2. Evaluation of drug-likeness, pharmacokinetics properties, and toxicity profiles.

Drug-likeness, pharmacokinetics properties, and toxicity profiles of the selected flavonoids were evaluated using three open-sources platforms; SwissADME, ADMETLab 2.0, and ProTox-II. The SwissADME (http://www.swissadme.ch/) was used to predict physiochemical descriptors, ADME parameters, and medicinal chemistry suitability [30]. ADMETLab 2.0 (https://admetmesh.scbdd.com/) was used to analyse comprehensive absorption, distribution, metabolism, and excretion (ADME) analysis. Meanwhile, toxicity predictions were generated using ProTox-II (https://tox-new.charite.de/protox_II/). Canonical SMILES for each compound were obtained from the PubChem database to support these assessments.

### 2.2. Structure refinement and validation

#### 2.2.1. Target receptor retrieval.

The PCSK9 crystal structure (PDB ID: 5OCA) was retrieved from the Protein Data Bank (https://www.rcsb.org/). This structure was selected because of its high crystallographic resolution (2.30 Å), which provides reliable atomic coordinates for structure-based drug discovery and well-defined binding pockets for molecular docking. Furthermore, the structure preserves the catalytic and C-terminal V-domains of PCSK9, which are directly involved in ligand recognition and are therefore suitable for identifying potential small-molecule binding sites. The complete PCSK9 amino acid sequence was obtained from the UnitProtKB database (https://www.uniprot.org/) to identify the region of local similarity between the target protein and crystal structure [31]. The crystal structure was first visualized and structurally inspected using PyMOL version 2.5.4 [32]. Prior to refinement, crystallographic water molecules were removed from the crystal structure. Chains C and D, corresponding to the crystallized antibody, were also removed, whereas PCSK9 chains A and B, HSPG, and the associated sodium ion were retained because the docking study was designed to evaluate flavonoid binding within the biologically relevant PCSK9-HSPG complex. HSPG has been demonstrated to function as a physiological co-receptor that captures PCSK9 and facilitates subsequent PCSK9-LDLR complex formation [33]. Alignment of the UniProt sequence with the 5OCA crystal structure facilitated the identification of unresolved or missing residues, which were subsequently incorporated into the structural refinement process.

#### 2.2.2. Refinement of PCSK9.

The three-dimensional structure of human proprotein convertase subtilisin/kexin type 9 (PCSK9) was refined using the SWISS-MODEL server (https://swissmodel.expasy.org/) [34]. The experimentally determined high-resolution crystal structure of human PCSK9 (PDB ID: 5OCA) was selected as the structural template owing to its structural accuracy and suitability for structure-based drug discovery. The amino acid sequence of human PCSK9 retrieved from the UniProt database was aligned with the template sequence to reconstruct missing residues and improve the completeness of the protein model prior to molecular docking.

Model refinement was performed using the ProMod3 modelling engine implemented in SWISS-MODEL. During refinement, missing residues and side chains were rebuilt based on the template structure, followed by side-chain optimization and stereochemical correction. The refined model subsequently subjected to energy minimization using the GROMOS96 force field to relieve steric clashes, optimize atomic geometry, and improve the overall structural stability of the protein [35].

#### 2.2.3. Validation of protein.

The generated model of refinement PCSK9 and native structure of PCSK9 were downloaded as PDB files, and structural alignment was performed using PyMOL Version 2.5.4 [32] to calculate the Root Mean Square Deviation (RMSD). This is to provide a quantitative measure of model accuracy and validation [36]. The analysis identified the optimal chain alignment that best matched to the template structure. Further assessment of the refinement PCSK9 internal consistency and reliability was conducted using the UCLA-DOE LAB SAVES server (Version 6.1) (https://saves.mbi.ucla.edu/), incorporating ERRAT, VERIFY3D, and PROCHECK analyses.

### 2.3. Molecular docking

Molecular docking was performed using AutoDock Vina Version 1.1 [37]. The target protein and ligand structures were initially loaded in PDB format using AutoDock Tools (ADT) and converted to the PDBQT format required, a readable format for AutoDock Vina. Using the same tools, the ligand structure was subjected to energy minimization (EM).

Docking was carried out in two stages; an initial blind docking step, followed by targeted docking. The purpose of blind docking is to identify the location of pocket for the ligands across the entire protein surface. Meanwhile, target docking focused on the consensus region identified during blind docking, representing the biologically relevant active site. For blind docking, the grid box dimension using ADT workspace were set to X: 80 Å, Y:84 Å, and Z: 64 Å with a center of X: −50.092, Y: −1.065, Z: −22.288. Meanwhile, for targeted docking, the grid box was set to X: 40 Å, Y: 40 Å, Z: 40 Å with center X: −58.78, Y: −14.904, and Z: −9.095. The exhaustiveness parameter was maintained at the default value of 8. The interaction between the best-docked protein-ligand complexes were subsequently analyzed using BIOVIA Discovery Studio to visualize hydrogen bonding, hydrophobic interactions, and other non-covalent contacts.

### 2.4. Molecular dynamic simulation

MD simulations of the best-docked protein–ligand complexes, together with the FDA-approved drug simvastatin as the reference compound, were performed using GROMACS 2020.6 [38]. Ligand topology files were generated using ACPYPE to convert the AMBER parameters into a GROMACS-compatible format, while the protein topology was generated using the AMBER99SB force field [39]. Each protein–ligand complex was solvated in a cubic box containing TIP3P water molecules. The systems were neutralized by adding Na⁺ and Cl^-^ions, which were randomly distributed throughout the solvent. Energy minimization was carried out using the steepest descent algorithm until convergence was achieved to remove steric clashes and obtain a stable starting configuration [40].

Following energy minimization, each system was subjected two sequential equilibration phases with positional restraints applied to the protein heavy atoms. Thermal equilibration was first performed under the constant number of particles, volume, and temperature (NVT) ensemble for 100 ps at 310 K using the velocity-rescale (V-rescale) thermostat. This was followed by pressure equilibration under the constant number of particles, pressure, and temperature (NPT) ensemble for 100 ps at 1 bar using the Berendsen barostat to stabilize the system density. A cutoff distance of 1.2 nm was applied for van der Waals and short-range electrostatic interactions, whereas long-range electrostatic interactions were calculated using the Particle Mesh Ewald (PME) method [41]. Production molecular dynamics simulations were subsequently performed for 100 ns at 310 K in triplicate.

Trajectory analyses included structural stability parameters (root mean square deviation [RMSD], radius of gyration [Rg], and solvent-accessible surface area [SASA]) and structural flexibility parameters (root mean square fluctuation [RMSF], pocket volume, and hydrogen bond [H-bond] interactions).

Binding free energies of the protein-ligand complexes were estimated using the Molecular Mechanics Poisson-Boltzmann Surface Area (MM/PBSA) approach to quantify interaction strength and evaluate complex stability during molecular dynamics simulations. MM/PBSA calculations were performed using gmx_MMPBSA v1.6.3 on the equilibrated 70–100 ns region of the molecular dynamics trajectories. A total of 31 trajectory snapshots, extracted at 100 ps intervals, were used for the binding free energy calculations. The internal (solute) and external (solvent) dielectric constants were set to the default values of 1.0 and 80.0, respectively. The total binding free energy (ΔG_bind) was calculated as the sum of the molecular mechanics energy contributions, including van der Waals (ΔE_vdW) and electrostatic (ΔE_ele) interactions, together with the solvation free energy contributions, comprising polar (Poisson–Boltzmann; ΔG_polar) and non-polar (ΔG_non-polar) solvation energies.

## 3. Results

A summary of the findings is presented in Fig 2. The workflow illustrated the screening of 29 compounds through four sequential steps: toxicity screening, structure refinement, molecular docking and molecular dynamics simulation.

**Fig 2:**
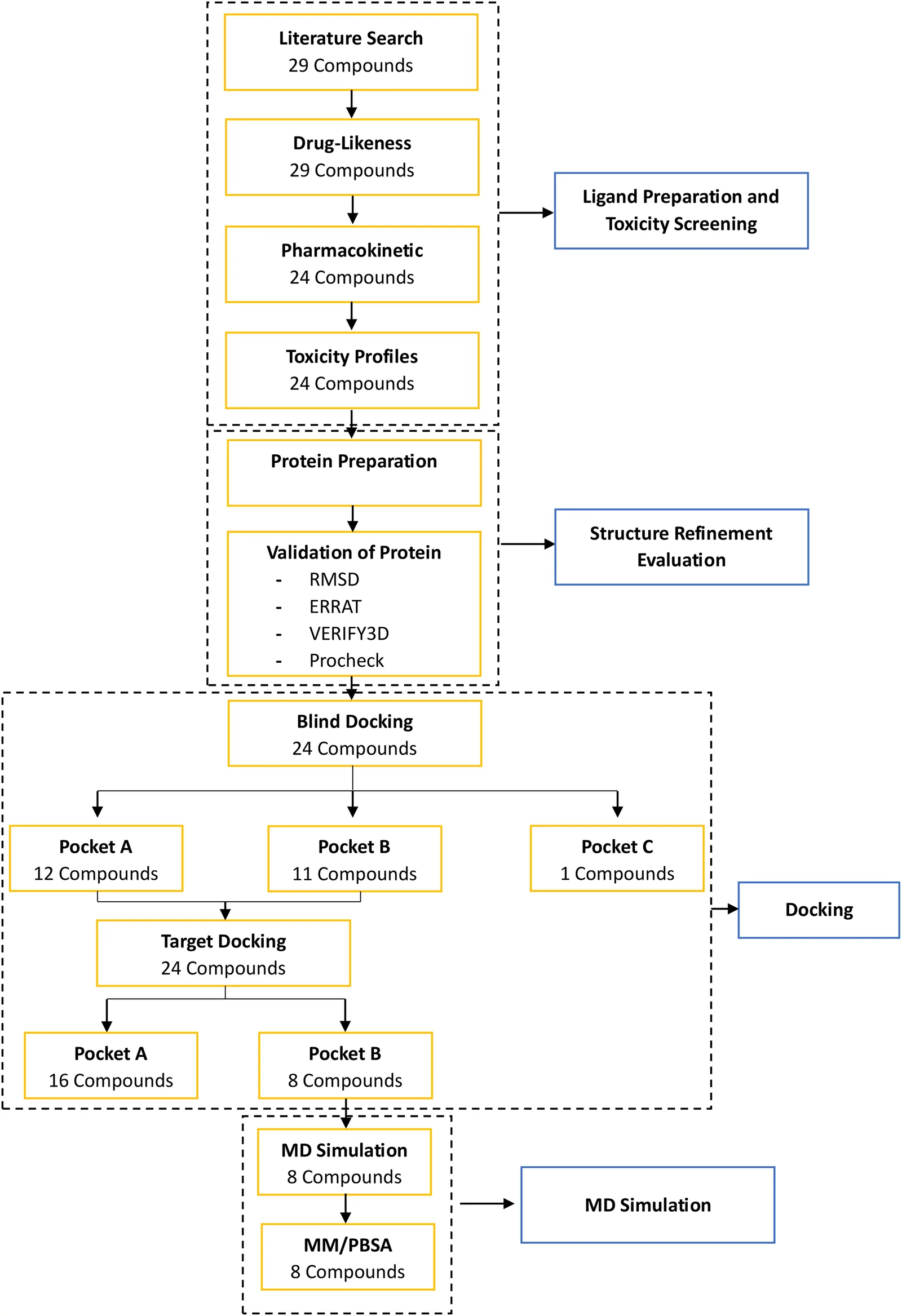
Workflow of findings potential flavonoids. The workflow shows the sequential screening and selection of flavonoids based on drug-likeness, pharmacokinetic and toxicity profiles, molecular docking, molecular dynamics simulation, and MM/PBSA analysis.

### 3.1. Ligand preparation and toxicity screening

#### 3.1.1. Drug-likeness.

Drug-likeness of 29 compound (28 flavonoids and one FDA-approved drug) were evaluated using SwissADME. Based on Lipinski’s Rule of Five, 24 compounds met all criteria as shown in S1 Table indicating favourable molecular properties, good predicted permeability, and acceptable oral absorption. Five flavonoids (Apigenin-7-O-neohesperidoside, Baicalin, Hesperidin, Rutin, and Theaflavin) have not achieved and violated most the parameter suggesting limited drug-likeness. The 24 compounds that passed Lipinski “Rule of 5” were then proceed for further investigate.

#### 3.1.2. Pharmacokinetics properties.

Pharmacokinetic profiling (ADME) of the flavonoids (S2 Table) revealed that Tilianin the only compound shown to have positive human intestinal absorption, while most flavonoids demonstrated acceptable Caco-2 cell permeability. Five flavonoids (Apigenin, Daidzein, Diosmetin, Formononetin, and Tilianin) were predicted to be P-glycoprotein substrates. Distribution analysis showed that Eupatorin, Sinensetin, and Tilianin had plasma protein binding below 90%, and the remaining compounds demonstrated acceptable volumes of distribution. Most flavonoids displayed favourable interaction profiles with CYP1A2, CYP2C19, CYP2C9, CYP2D6, and CYP3A4, indicating a low risk of major metabolic inhibition. Excretion analysis indicated generally longer predicted half-lives compared with simvastatin, and predominantly moderate renal clearance values. Overall, the pharmacokinetic assessment suggests that the majority of flavonoids possess acceptable ADME properties, leading to the selection of these 24 compounds for further investigation.

#### 3.1.3. Toxicity profiles.

Computational toxicity assessment of flavonoids was performed using ADMETLab 2.0 and ProTox-II to evaluate multiple endpoints, including hERG inhibition, hepatotoxicity, Ames mutagenicity, respiratory toxicity, carcinogenicity, skin sensitivity, and drug-induced liver injury. All flavonoids passed hERG and hepatotoxicity predictions, except simvastatin as shown in S3 Table. Ten compounds were predicted to be Ames-positive, suggesting potential mutagenic risk. ProTox-II analysis as as shown in S4 Table classified Fisetin, Myricetin, and Quercetin as Class III (toxic if swallowed), Naringenin, Pinocembrin, Simvastatin, and Taxifolin as Class IV (harmful if swallowed), and the remaining flavonoids as Class V (may be harmful), while Catechin and Gallocatechin were to be non-toxic (Class VI). Collectively, the toxicity assessment indicated that most of the 24 compounds possess acceptable safety profiles, supporting their potential as safe anti-hyperlipidemic candidates for further investigation.

### 3.2. Structure Refinement and Validation

#### 3.2.1. Refinement of PCSK9 Structure.

Refinement of the PCSK9 3D protein structure was performed using SWISS-MODEL by employing amino acid FASTA sequence of PDB ID: 5OCA retrieved from the Protein Data Bank. The model was constructed based on a template with 62% sequence similarity. Structural quality assessment using the QMEAN-Z score, yielded a value of −1.14, reflecting the degree of nativeness of the modelled structure. This score falls within the generally accepted range of> −4.0 indicating that the refined PCSK9 model is reliable and acceptable [42,43]. The refined 3D structure of PCSK9 is shown in Fig 3 and is hereafter referred to as ‘refinement PCSK9’ throughout this manuscript.

**Fig 3:**
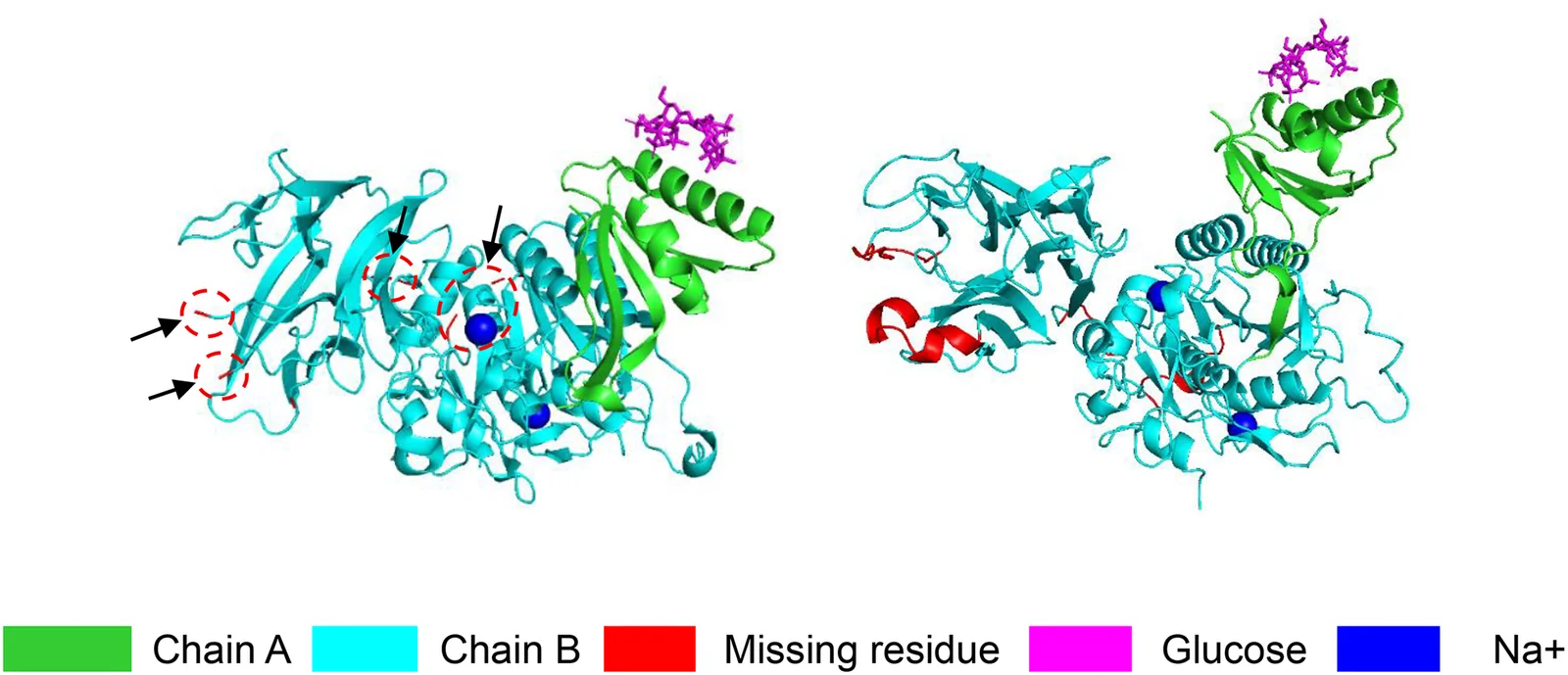
Comparison of the PCSK9 structures before and after refinement. The left panel shows the PCSK9 structure with a missing residue, whereas the right panel shows the refined PCSK9 structure.

#### 3.2.2. Structural Validation.

The refined PCSK9 structure was align with the native protein, revealed a root-mean square deviation (RMSD) of 0.075 Å, which is within the accepted threshold of 2 Å, and is indicative of successful structural refinement [44]. Model quality assessment was further evaluated using ERRAT, VERIFY3D, and PROCHECK (S5 Table). The refined model achieved an ERRAT score of 91.65%, while VERIFY3D indicated 67.85% of residues had an average 3D-1D score >0.1. Ramachandran plot analysis showed that 90.5% of residues were located in the most favored regions (See S1 Fig). Given that high-quality models typically exhibit ERRAT scores >50% [45] and PROCHECK assessments favor models with ≥90% of residues in most favored regions [46]. Overall, these validation results collectively indicate that the refined PCSK9 model is of high quality and suitable for further computational studies.

### 3.3. Docking of flavonoids

Molecular docking was performed by using the refined PCSK9 structure. The refined PCSK9 structure contains specific amino acid residues that preferentially interact with flavonoids, influencing their pharmacological activity. Molecular docking was carried out in two conditions; blind and targeted docking. Blind docking identified three potential binding pockets as illustrated in Fig 4. Twelve ligands with binding affinity ranging from −8.7 to −10.2 kcal/mol bind to pocket A. Meanwhile, eleven ligands with binding affinity ranging from −7.1 to −8.7 kcal/mol bind to pocket B, and only one ligand (−6.8 kcal/mol) which is FDA approved drug (Simvastatin) bind to pocket C.

**Fig 4:**
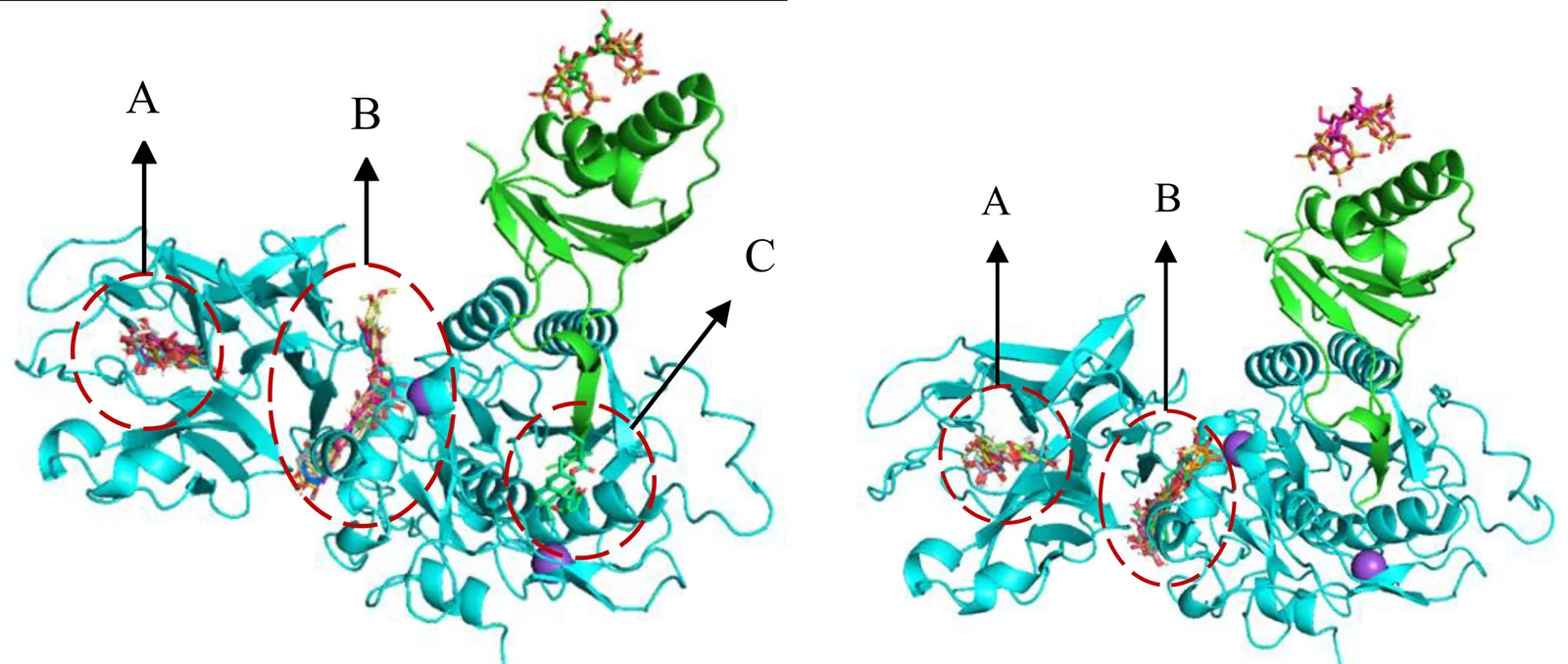
Blind docking (left) and target docking (right) of 24 compounds (23 flavonoids and one FDA-approved drug Simvastatin) with PCSK9. (Left) Blind docking identified three binding pockets (A-C) among 24 compounds: Pocket A (12 ligands: Apigenin, Catechin, Cyanidin, Daidzein, Delphinidin, Diosmetin, Eupatorin, Gallocatechin, Luteolin, Petunidin, Taxifolin, Tilianin), Pocket B (11 ligands: Baicalein, Epiafzelechin, Fisetin, Formononetin, Isorhamnetin, Kaempferol, Myricetin, Naringenin, Pinocembrin, Quercetin, Sinensetin), and Pocket C (Simvastatin as control). (Right) Target docking evaluated compounds within pockets A and B. Simvastatin was included as the reference compound: Pocket A (16 ligands: Apigenin, Baicalein, Catechin, Cyanidin, Daidzein, Delphinidin, Epiafzelechin, Fisetin, Formononetin, Gallocatechin, Kaempferol, Luteolin, Naringenin, Pinocembrin, Quercetin, Taxifolin) and Pocket B (8 ligands: Diosmetin, Eupatorin, Isorhamnetin, Myricetin, Petunidin, Sinensetin, Tilianin, Simvastatin).

Targeted docking was performed on two predicted binding pockets, A and B, resulting in 16 and 8 ligands binding to Pocket A and Pocket B, respectively. Simvastatin, an FDA-approved lipid-lowering drug, was used as the reference compound for docking comparison and was found to bind Pocket B with a binding affinity of −9.1 kcal/mol, forming two hydrogen bonds with Leu436 and Asn439. Among the flavonoids docked to Pocket B, Tilianin exhibited the strongest binding affinity (−10.2 kcal/mol), followed by Myricetin, Petunidin, Sinensetin, Isorhamnetin, Eupatorin, and Diosmetin, with binding affinities ranging from −8.5 to −10.2 kcal/mol, demonstrating comparable or more favourable binding than the reference compound. The flavonoids formed hydrogen bond interactions with distinct amino acid residues: Diosmetin (Val435), Eupatorin (Trp461), Isorhamnetin (Thr459 and Arg525), Myricetin (Glu426, Arg434, Val435, and Thr459), Petunidin (Thr459, Trp461, and Ala649), Tilianin (Asp360, Leu436, Arg306, Thr459, Trp461, and Asp651), and Sinensetin (Asp651), as shown in Table 2. Given the limitations of molecular docking in predicting binding stability and dynamic protein-ligand interactions, molecular dynamics simulations were subsequently performed to evaluate complex stability, interaction persistence, and ligand-induced conformational changes, thereby enabling a more comprehensive comparison between the flavonoids and simvastatin.

**Table 2:** The blind and target docking of the 24 flavonoids and hydrogen bond interactions with PCSK9.

					Residue Amino Acid Interactions
	Ligand	Blind Docking Binding Energy (kcal/mol)	Target Docking Affinity (kcal/mol)	Conventional H-bond	Carbon Hydrogen bond, Metal Acceptor, Unfavourable Donor-Donor, Unfavourable Acceptor-Acceptor, Pi-Donor Hydrogen Bond, Pi-Pi T-shaped, Pi-Sigma, Pi-cation, Amide-Pi Stacked, Alkyl, Pi-Alkyl, Pi-Anion
1.	Apigenin	−9.7	−9.7	Ser488, Arg495, Cys562, Ser636, Gly640, Thr641	Ser487, Glu498, Ser564, Ala637, Pro639, Val644, Ala647
2.	Baicalein	−8.5	−9.3	Ser636, Ala637	Trp566, Pro639, Val644
3.	Catechin	−9.8	−9.8	Ser488, Arg495, Cys562, Ser636, Gly640, Thr641, Ala647	Glu498, Ala637, Pro639
4.	Cyanidin	−9.1	−9.4	Arg495, Cys562, Ser636, Leu638, Gly640, Thr641, Ala647	Ser448, Glu498, Ala637, Pro639
5.	Daidzein	−8.7	−9.3	Arg495, Cys635, Gly640, Thr641	Glu498, Cys562, Ser564, Ser636, Ala637, Pro639, Ala647
6.	Delphinidin	−9.6	−9.3	Ser488, Arg495, Cys562, Ser636, Thr641, Ala647	Ser487, Glu498, Ala637, Pro639
7.	Diosmetin	−10.0	−8.8	Val435	Pro331, Cys358, Thr437, Pro438, Arg458, Val460, Trp461, Na701
8.	Epiafzelechin	−8.1	−8.8	Ser488, Ser636, Ala637	Glu498, Pro639, Ala647
9.	Eupatorin	−10.1	−8.7	Trp461	Pro331, Ile416 Thr437, Pro438, Leu440, Val460, Val650
10.	Fisetin	−8.5	−9.5	Ser488, Arg495, Leu638, Gly640, Thr641, Ala647	Glu498, Ser564, Ala637, Pro639
11.	Formononetin	−8.1	−9.2	Cys486, Arg495, Cys635, Gly640, Thr641	Glu498, Cys562, Ser564, Leu571, His591, Ser636, Ala637, Pro639, Ala647
12.	Gallocatechin	−9.7	−9.6	Ser488, Arg495, Cys562, Ser636, Thr641	Ser487, Glu498, Ser563, Ala637, Pro639, Gly640, Ala647
13.	Isorhamnetin	−8.2	−8.5	Thr459, Arg525	Ile416, Pro438, Trp461, Val650
14.	Kaempferol	−8.7	−9.8	Ser488, Arg495, Cys562, Ser636, Gly640, Thr641	Ser487, Glu498, Ser564, Ala637, Pro639, Ala647
15.	Luteolin	−9.8	−9.8	His591, Ser636, Ala637, Ser642, Val644	Trp566, Leu571, Pro639
16.	Myricetin	−8.5	−8.7	Glu426, Arg434, Val435, Thr459	Leu440, Val650, Asp651
17.	Naringenin	−7.8	−9.7	Ser488, Arg495, Cys562, Ser636, Thr641, Ala647	Glu498, Ser564, Pro639, Val644
18.	Petunidin	−9.4	−8.5	Thr459, Trp461, Ala649	Ile416, Val435, Pro438, Val650
19.	Pinocembrin	−8.3	−9.3	Ser488, Arg495, Cys562, Ser636, Ala647	Glu498, Pro639
20.	Quercetin	−8.4	−9.9	Ser488, Arg495, Glu498, Cys562, Ser636, Gly640, Thr641, Ala647	Ser564, Ala637, Pro639
21.	Simvastatin (Control)	−6.8	−9.1	Leu436, Asn439	Asp360, Ile416, Lys421, Leu440
22.	Sinensetin	−7.1	−7.9	Asp651	Arg357, Asp360, Ile416, Asp422, Val435, Leu436, Thr437, Pro438, Trp461, Tyr648, Val650
23.	Taxifolin	−10.2	−10.2	Ser488, Arg495, Cys562, Gly640, Thr641	Ser487, Glu498, Ser564, Ala637, Pro639, Ala647
24.	Tilianin	−10.1	−10.2	Asp360, Leu436, Arg458, Thr459, Trp461, Asp651	Ile416, Val435, Pro438, Val650

### 3.4. Molecular dynamics simulation

Molecular dynamics simulations were performed for eight PCSK9-ligand complexes. Each system was simulated in triplicate to ensure reproducibility, demonstrating consistent results that indicate stable complexes and reliable binding interactions (S6 Table). The simulation trajectories were systematically analysed to evaluate the complex stability, flexibility and molecular interactions.

#### 3.4.1. Structural stability.

Structural stability of PCSK9-ligand complexes was examined. The root means square deviation (RMSD) analysis of all complexes indicated that the protein backbone remained stable throughout the simulation reaching equilibrium at 70 ns (Fig 5). PCSK9-ligand complexes exhibiting slightly higher deviation than Simvastatin (control) (See Table 3). Radius of gyration (Rg) values demonstrated similar compactness across all complexes with Eupatorin and Tilianin showing slightly lower Rg than the control, suggesting enhanced structural stability. Solvent-accessible surface area (SASA) analysis further confirmed minimal protein expansion and consistent hydrophobic core stability in all complexes. Overall, RMSD, Rg, and SASA analyses collectively indicate that the PCSK9-ligand complexes maintain stable conformations compare to the control, supporting their potential suitability for further computational and pharmacological studies.

**Table 3:** Analysis of MD simulation of FDA approved drug Simvastatin (control) and flavonoids.

Results		Simvastatin (Control)	Diosmetin	Eupatorin	Isorhamnetin	Myricetin	Petunidin	Sinensetin	Tilianin
Molecular Docking	Target Docking score (kcal/mol)	−9.1	−8.8	−8.7	−8.5	−8.7	−8.5	−7.9	−10.2
MD Simulation Stability	Average RMSD backbone (nm)	0.2168	0.2537	0.2228	0.2470	0.2261	0.2253	0.2611	0.2224
	Average radius of gyration (nm)	2.6261	2.6312	2.6242	2.6629	2.6390	2.6349	2.6385	2.6221
	Average SASA protein (nm2)	265.3711	263.5167	263.2590	263.0578	262.8983	261.5920	263.5216	262.9896
MD Simulation Flexibility	Average RMSF (nm)	0.1439	0.1333	0.1361	0.1295	0.1408	0.1335	0.1361	0.1298
	Average RMSD Pocket (nm)	33.83	34.06	39.66	32.96	35.75	32.65	32.41	37.47
	Average number of protein compound hydrogen bond (>50%)	3	1	1	2	2	2	2	1
	Binding free energy calculation MM/PBSA (kcal/mol)	−30.41	−15.07	−18.64	−17.86	−28.09	−18.24	−19.09	−29.18

**Fig 5:**
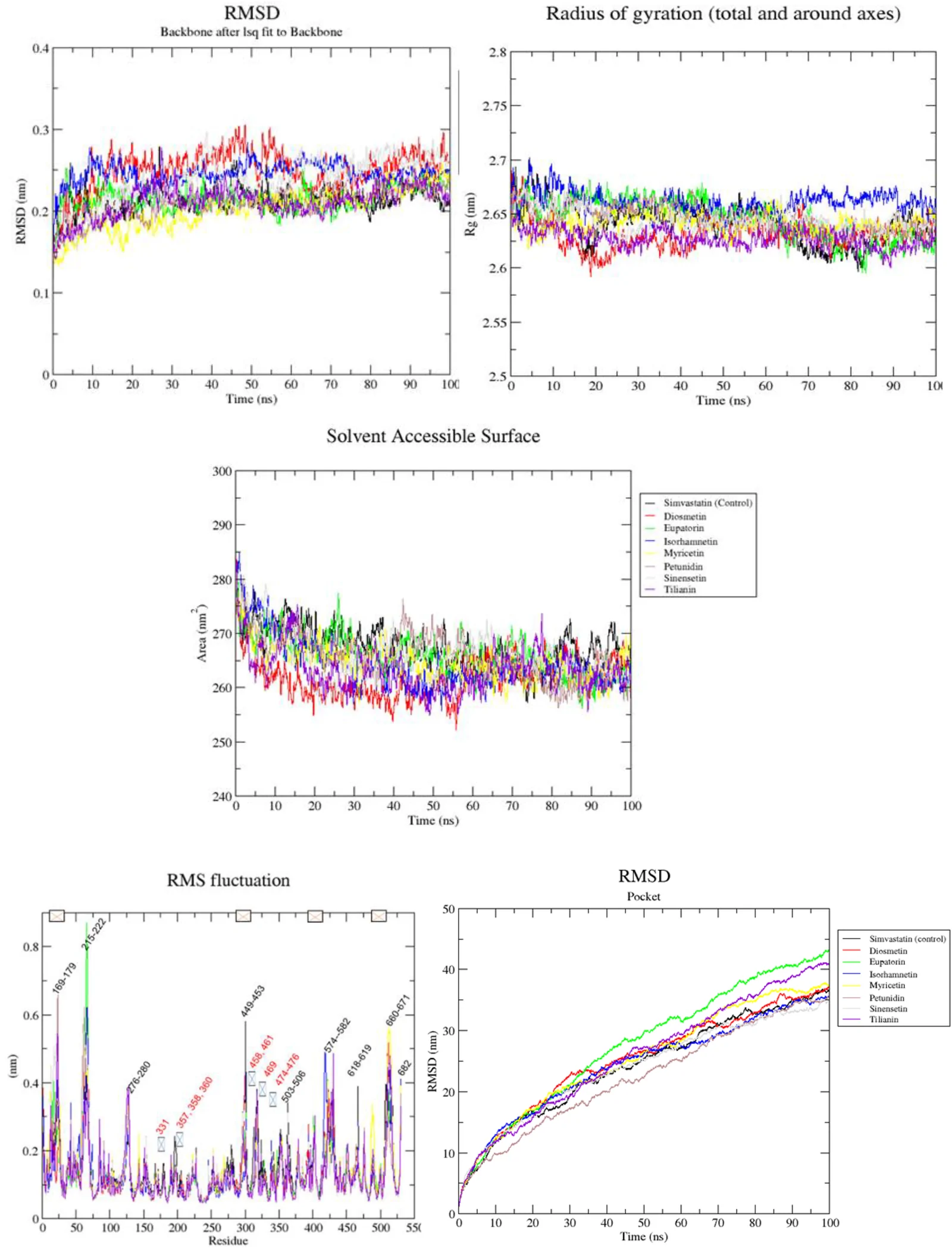
Molecular dynamics stability analysis of PCSK9-ligand complexes. The plots show backbone RMSD (top left), radius of gyration (top right), solvent-accessible surface area (centre), RMSF (bottom left), and pocket RMSD (bottom right).

#### 3.4.2. Structural flexibility.

Root mean square fluctuation (RMSF) analysis was performed to evaluate the flexibility of individual amino acid residues in the PCSK9-ligand complexes throughout the molecular dynamics simulation. All eight flavonoid bound complexes exhibited RMSF profiles comparable to the Simvastatin control, with pronounced fluctuations observed at residues 169–179, 215–222, 276–280, 449–453, 469, 503–506, 574–582, 618–619, 660–671, and 682 (Fig 5). These highly flexible regions predominantly correspond to surface-exposed loop segments, including residues reconstructed during protein refinement. Isorhamnetin, Myricetin, Petunidin, Sinensetin, and Tilianin exhibited slightly higher residue fluctuations than Simvastatin, Diosmetin, and Eupatorin. Importantly, the regions displaying the greatest flexibility were located away from the ligand-binding site, indicating that ligand binding did not destabilize the active site of PCSK9.

The relatively low RMSF values observed around the binding pocket suggest that the ligands effectively stabilized the local protein conformation through favorable non-covalent interactions. Hydrogen bonding, together with van der Waals and hydrophobic interactions, likely restricted local residue mobility and contributed maintain the structural integrity of the PCSK9-ligand complexes throughout the simulation. This interpretation is supported by the hydrogen bond occupancy analysis that was discussed in the next section (3.4.2.1) which demonstrated persistent interactions between the ligands and key binding-site residues, thereby reinforcing complex stability during the simulation.

Pocket volume analysis further demonstrated only minor differences among the ligand-bound complexes, with binding pocket volumes ranging from 75.64 to 76.19 nm^3^ compared with 76.55 nm^3^ for Simvastatin (Fig 5). The slight reduction in pocket volume following ligand binding suggests subtle conformational adaptation of the binding cavity to accommodate the ligands without inducing major structural rearrangements. Collectively, the RMSF and pocket volume analyses indicate that all ligands maintained stable binding within the PCSK9 active site while preserving the overall structural integrity of the protein.

**3.4.2.1. Hydrogen bond analysis:** Further investigation through hydrogen bond occupancy analysis identifies key interacting residues in catalytic and V domain, and the duration of interactions (Table 4). A 50% occupancy of hydrogen bonds is regarded as noteworthy. Simvastatin engaged to residue that involve in catalytic domain, Leu436, Asn439, and V domain, Trp461 with high occupancy 82.2%, 64.2% and 64.4%, respectively (Fig 6(a)). Meanwhile, Diosmetin interact with Thr459 at V domain with 90.3% occupancy (Fig 6(b)), followed by Eupatorin interact with residue Leu436 at catalytic domain (50.2%) (Fig 6(c)). Isorhamnetin was found to interact with catalytic domain Glu426 (50.7%) and V domain Trp461 (75.0%) (Fig 6(d)), Myricetin interact with catalytic domain Val435 (53.1%) and V domainTrp461 (75.6%) (Fig 6(e)), Petunidin form interaction with catalytic domain Leu436 (90.8%) and V-domain Trp461 (52.0%) (Fig 6(f)), Sinensetin interact with V domain Thr459 (68.8%) and Asp651 (50.2%) (Fig 6(g)), and Tilianin was found to interact with V domain Arg458 (94.3%) (Fig 6(h)). Notably, Petunidin and Eupatorin shared catalytic domain Leu436 as a common hydrogen bonding residue with Simvastatin, exhibiting the highest occupancy among all ligands. These findings indicate that hydrogen bonding contributes substantially to the stability of the PCSK9-ligand complexes.

**Table 4:** List of hydrogen bond occupancy of ligands to refinement PCSK9 residues.

Residues	Hydrogen bond occupancy (%)							
	Simvastatin (Control)	Diosmetin	Eupatorin	Isorhamnetin	Myricetin	Petunidin	Sinensetin	Tilianin
Asp360**	0	0.7	0	0	0	0	0	32.5
Glu426**	0	0	0	50.7	48.8*	15.4	0	0
Val435**	5.1	24.4*	9.5	0	53.1*	0	0	0
Leu436**	82.2*	0	50.2	0	0	90.8	0	21.9*
Asn439**	64.2*	34.0	13.1	0	40.9	1.6*	12.0	41.5
Arg458***	0	0	0	0	0	0	0	94.3*
Thr459***	0	90.3	0	3.4*	49.7*	0.4*	68.8	4.0*
Trp461***	64.4	15.3*	3.6*	75.0*	75.6	52.0*	0.3	0.3*
Arg525***	0	0	0	0	0	0	0	0
Ala649	0	0	0	41.7	0	28.7*	0	0
Asp651***	0	19.3	0	0	0	0	50.2	5.8*

**Catalytic domain

***V-domain

*Hydrogen bond from docking

**Fig 6:**
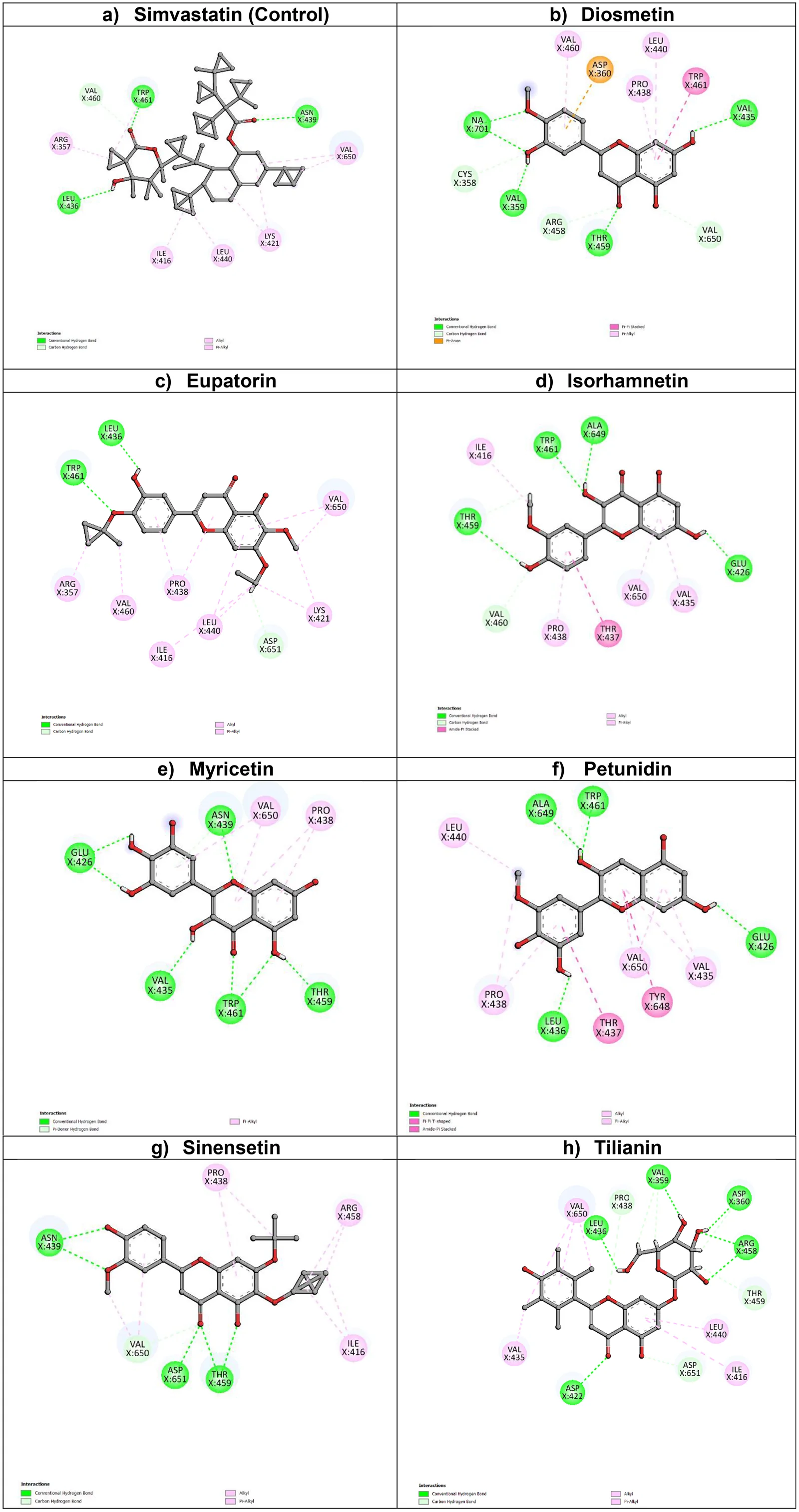
Representative two-dimensional interactions of ligands with PCSK9 during molecular dynamics simulation. (a) Simvastatin, (b) Diosmetin, (c) Eupatorin, (d) Isorhamnetin, (e) Myricetin, (f) Petunidin, (g) Sinensetin, and (h) Tilianin.

#### 3.4.3. Binding free energy calculation.

Binding free energies of the protein-ligand complexes were estimated using MM-PBSA over the stabilized period of the MD simulation (70−100 ns) (Fig 7). Simvastatin (control) exhibited the strongest binding, with a ΔG_bind of −30.41 kcal/mol. Among the flavonoids, Tilianin displayed the most favorable binding (−29.18 kcal/mol), followed by Myricetin (−28.09 kcal/mol), Sinensetin (−19.09 kcal/mol), Eupatorin (−18.64 kcal/mol), Petunidin (−18.24 kcal/mol), Isorhamnetin (−17.86 kcal/mol), and Diosmetin (−15.07 kcal/mol). Residue-level energy decomposition indicated that all ligands formed substantial interactions with key PCSK9 residues, underscoring their potential as effective PCSK9 inhibitors. Further MM-PBSA decomposition analysis revealed that van der Waals interactions were the primary contributors to binding free energy across all complexes, emphasizing the role of hydrophobic and shape-complementary interactions (S7 Table). Electrostatic interactions contributed significantly for ligands bearing multiple polar functional groups, while polar solvation energies opposed binding. Consequently, the favorable binding free energies observed result from a balance between strong molecular mechanics interactions and unfavorable solvation effects.

**Fig 7:**
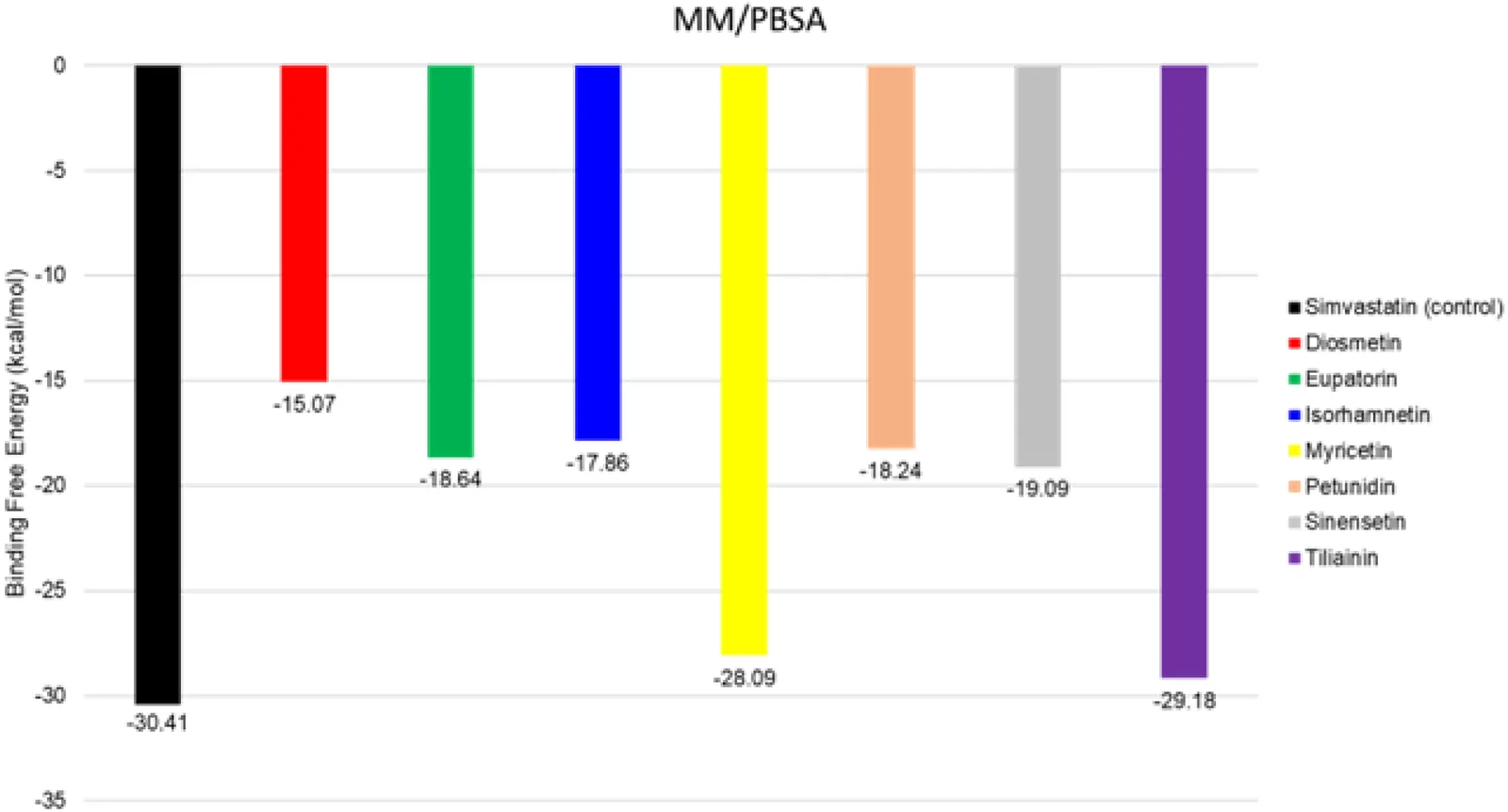
MM/PBSA binding free energy of ligands complexed with refined PCSK9. The graph shows the calculated total binding free energy of simvastatin and the selected flavonoids based on equilibrated molecular dynamics simulations.

## 4. Discussion

In this study, we employed an integrative computational approach to evaluate flavonoids derived from natural products as potential therapeutic inhibitors of PCSK9 for the reduction of cholesterol levels. Extensive evidence from the literature indicates that therapeutic inhibition of PCSK9 represents an effective strategy for managing hypercholesterolemia and reducing cardiovascular disease (CVD) risk. PCSK9 plays a pivotal role in cholesterol homeostasis by promoting lysosomal degradation of the low-density lipoprotein receptor (LDLR), thereby limiting hepatic LDL-C clearance and contributing to elevated circulating low-density lipoprotein cholesterol levels [47]. Monoclonal antibody such as PCSK9 inhibitors have demonstrated substantial LDL-C lowering efficacy and favourable safety profiles in both familial hypercholesterolemia (FH) and non-FH populations. However, despite their clinical effectiveness, widespread implementation remains constrained by high costs, limited accessibility, and restrictive prescription criteria that prioritize high-risk or FH patients [48,49]. This disparity highlights a critical unmet need for affordable, orally available PCSK9-targeting agents that could broaden therapeutic access and improve population-level cardiovascular outcomes.

Flavonoids have attracted considerable attention due to their multiple beneficial effects on cardiovascular health. For example, Diosmetin has been reported to exhibit strong cholesterol-lowering activity in in-vitro studies [50]. In addition, in-vivo evidence indicates that Sinensetin effectively reducing blood triglyceride and cholesterol levels, as well as hepatic lipid accumulation [51]. Beyond their lipid-lowering potential, flavonoids are widely recognized for their anti-inflammatory, antioxidant, and cardioprotective properties, which are highly relevant to the pathophysiology of atherosclerosis and cardiovascular disease [52]. Chronic inflammation and oxidative stress are key contributors to endothelial dysfunction and plaque progression, processes that are not fully addressed by lipid-lowering therapy alone [53,54]. Other study, also conclude Polymethoxyflavones from *Kaempferia parviflora* modulate adipocyte function and lipid metabolism, suggesting beneficial for cholesterol regulation, managing obesity and metabolic balance [55]. This could lead to oxidative stress, hypertrophy, inflammatory, and cellular dysfunction. Previous studies have demonstrated that kaempferol can modulate inflammatory signalling pathways, reduce oxidative stress, and improve vascular function, suggesting their potential utility as multi-functional cardiovascular agents [56–58]. These pleiotropic effects provide a strong biological rationale for exploring flavonoids as PCSK9-targeting candidates that may offer complementary benefits beyond LDL-C reduction.

The flavonoids evaluated in this study demonstrated overall favourable drug-likeness, pharmacokinetic, and toxicity profiles. However, from an initial screening of 29 compounds, seven flavonoids Diosmetin, Eupatorin, Isorhamnetin, Myricetin, Petunidin, Sinensetin, and Tilianin alongside one FDA-approved drug Simvastatin as a reference control, were selected based on favourable drug-likeness and pharmacokinetic profiles. Notably, several flavonoids exhibited predicted absorption and distribution parameters comparable to Simvastatin, supporting their potential as orally bioavailable agents. In addition, majority flavonoids were predicted to have a good stability and low toxicity, suggesting that natural flavonoids may represent promising lead compounds for the development of cost-effective small-molecule PCSK9 inhibitors that can benefit to all of the population. However, these predictions require further validation through computational and experimental validation before therapeutic potential can be established.

Molecular docking and molecular dynamics (MD) simulations provided mechanistic insights into the interaction patterns between flavonoids and PCSK9. Hydrogen bond analyses and RMSF profiles further confirmed the stability of these complexes, indicating that ligand binding did not induce destabilizing conformational fluctuations in PCSK9. This is an essential requirement for sustained inhibitory activity. A key observation was the dual-site binding behaviour exhibited by the compounds, involving both the catalytic domain and the C-terminal V-domain of PCSK9. Interactions within the catalytic domain particularly with residues such as Asp360, Glu426, Val435, Leu436, and Asn439 are notable, as this region has been reported to influence PCSK9 conformational dynamics and allosteric regulation [59]. In contrast, residues located within the V-domain including Arg458, Thr459, Trp461, Ala525, and Asp651, are directly implicated in PCSK9-LDLR binding, making them the primary determinants of functional inhibition [60,61].

The present findings demonstrate that all seven flavonoids engaged either one or both of these critical domains, with several compounds particularly Tilianin, Myricetin, and Sinensetin forming stable interactions with V-domain residues such as Arg458, Thr459, Trp461, and Asp651. These interactions closely mimicked those observed with Simvastatin and other reported PCSK9 inhibitors, suggesting that the flavonoids may effectively hinder PCSK9-LDLR binding. In contrast, the results reveal that Eupatorin is predicted to bind exclusively to the catalytic domain. This binding pattern suggests a potential role in modulating PCSK9 activity through an allosteric mechanism rather than direct competitive inhibition of LDLR binding. This hypothesis is supported by the exclusive interaction of Eupatorin with catalytic-domain residues, whereas flavonoids predicted to directly interfere with PCSK9-LDLR binding predominantly interacted with V-domain residues. Similar mechanisms have been proposed for other small-molecule and flavonoid-derived allosteric modulators of the PCSK9 structural network [62–64]. The polymethoxyflavone structure of Eupatorin, containing multiple methoxy substituents and a hydrophobic aromatic scaffold, may favour interactions within the catalytic pocket, contributing to its distinct binding preference. Although Eupatorin has not previously been investigated as a PCSK9 inhibitor, it has been widely reported to possess antioxidant, anti-inflammatory, vasodilatory, and hepatoprotective properties [65]. Previous studies have also demonstrated that Eupatorin improves endothelial function through nitric oxide-mediated vasorelaxation, suggesting potential cardiovascular benefits [66]. The relevance of these binding sites is further strengthened by consistency with previous computational and experimental studies. Natural products derived from *Monanthotaxis filipes* have been reported to interact with residues such as Pro438, Cys358, Val460, and Trp461 [63], while carotenoids like astaxanthin target Asp360, Val460, and Trp461 [67]. Additionally, repurposed drugs including amikacin and natamycin form persistent hydrogen bonds with Asp360 and Arg458 [65]. Importantly, residues such as Pro331, Arg357, Cys358, Asp360, Arg458, and Arg476 have been identified as key allosteric regulators of PCSK9 activity [68]. While the V-domain of PCSK9 directly mediates LDLR binding and thus represents the primary site for functional inhibition, whereas interactions at the catalytic domain contributes indirectly through allosteric regulation by modulating protein conformation and stabilizing the PCSK9-LDLR interface.

Thermodynamic stability was further corroborated by MM/PBSA binding free energy analyses. Tilianin (−29.18 kcal/mol) and Myricetin (−28.09 kcal/mol) exhibited binding affinities comparable to Simvastatin (−30.41 kcal/mol), highlighting their strong interaction potential. Energy decomposition analyses revealed that van der Waals interactions and electrostatic forces were the dominant contributors to complex stability. This observation is consistent with the planar, polyphenolic architecture of flavonoids, which facilitates favourable hydrophobic packing and π–π interactions within PCSK9 binding pockets. The persistence of hydrogen bonding at key residues at catalytic and V-domain also targeted by established inhibitors further supports a robust and reproducible binding mode.

Taken together, these computational results suggest that selected flavonoids, particularly Tilianin and Myricetin, possess strong structural, energetic, and dynamic characteristics consistent with effective PCSK9 inhibition. Flavonoids engaging the V-domain are likely to directly block PCSK9-LDLR interactions, whereas those restricted to the catalytic domain, such as Eupatorin, may influence protein conformation through allosteric modulation without directly inhibiting LDLR binding. While these computational findings are promising, experimental validation through in-vitro PCSK9-LDLR binding assays and subsequent in-vivo studies will be essential to confirm their functional efficacy and translational relevance. Nonetheless, this study provides a strong mechanistic and thermodynamic foundation supporting flavonoids as viable small-molecule candidates for the development of affordable and accessible PCSK9-targeting therapies. This research also aligns with Sustainable Development Goal 3 (SDG 3): Good Health and Well-Being, as it contributes to promoting health, improving well-being, and addressing health-related challenges.

## 5. Conclusion

In conclusion, this study systematically evaluated the potential of naturally occurring flavonoids as PCSK9 inhibitors using an integrated computational workflow. Of the 29 compounds initially screened, 24 compounds satisfied the drug-likeness and ADME criteria and demonstrated acceptable predicted toxicity profiles, supporting their suitability for further investigation. Based on molecular docking, seven flavonoids (Diosmetin, Eupatorin, Isorhamnetin, Myricetin, Petunidin, Sinensetin, and Tilianin) were selected for molecular dynamics simulations and MM-PBSA analyses. These compounds exhibited stable binding within the PCSK9 binding pocket, with most preferentially interacting with the V-domain involved in PCSK9-LDLR recognition. Hydrogen bonding together with van der Waals interactions contributed substantially to complex stability throughout the simulations. Among the investigated flavonoids, Tilianin demonstrated one of the most favorable binding profiles, with a docking score of 10.2 kcal/mol and an MM-PBSA binding free energy of −29.18 kcal/mol, comparable to Simvastatin (−30.41 kcal/mol). Overall, Tilianin, Myricetin, Sinensetin, Petunidin, Isorhamnetin, and Diosmetin emerged as the most promising candidates for PCSK9 inhibition and warrant further experimental validation. These findings provide a molecular basis for the development of flavonoid-based PCSK9 inhibitors and support subsequent in-vitro and in-vivo studies to confirm their therapeutic potential for hypercholesterolemia.

## Supporting information

S1 Fig Superimposition native structure of PCSK9 with refinement PCSK9 (left), and Ramachandran plot of refinement PCSK9. (DOCX)

S1 Table Drug-likeness data of the following flavonoids along with FDA-approved medicine. (DOCX)

S2 Table ADME Properties data of the following flavonoids and FDA-approved medicine. (DOCX)

S3 Table Toxicity prediction of the flavonoids and Simvastatin by using FDA-approved medicine. (DOCX)

S4 Table Toxicity Prediction of the flavonoids and FDA-approved medicine using ProTox II. (DOCX)

S5 Table Validation results of refinement PCSK9. (DOCX)

S6 Table Statistical analysis of RMSD backbone in triplicate data for all MD simulation. (DOCX)

S7 Table Decomposition of MM/PBSA binding free energy components for PCSK9-ligand complexes calculated from the equilibrated MD simulations. (DOCX)
