# Supplementary material for: Identification of flavonoids from natural products against hyperlipidaemia through computational approaches
Source: PLoS One. 2026 Oct 9;21(10):e0346073. doi: 10.1371/journal.pone.0346073 (PMC13652269; doi:10.1371/journal.pone.0346073)
Supplement: Supplementary file 1 [file pone.0346073.s001.docx]

**Fig S1.** Superimposition native structure of PCSK9 with refinement PCSK9 (left), and Ramachandran plot of refinement PCSK9.
