# Supplementary material for: Identification of flavonoids from natural products against hyperlipidaemia through computational approaches
Source: PLoS One. 2026 Oct 9;21(10):e0346073. doi: 10.1371/journal.pone.0346073 (PMC13652269; doi:10.1371/journal.pone.0346073)
Supplement: Supplementary file 2 [file pone.0346073.s002.docx]

**Table S1.** Drug-likeness data of the following flavonoids along with FDA-approved medicine.

|  | **Flavonoids** | **Molecular Weight** | **HBA** | **HBD** | **Consensus Log P** | **GI Absorption** | **Violation** |
| --- | --- | --- | --- | --- | --- | --- | --- |
| 1. | Apigenin | 270.24 | 5 | 3 | 2.11 | High | 0 |
| 2. | Apigenin-7-O-neohesperidoside | 578.52 | 14 | 8 | -0.66 | Low | 3 |
| 3. | Baicalein | 270.24 | 5 | 3 | 2.24 | High | 0 |
| 4. | Baicalin | 446.36 | 11 | 6 | 0.22 | Low | 2 |
| 5. | Catechin | 290.27 | 6 | 5 | 0.85 | High | 0 |
| 6. | Cyanidin | 287.24 | 6 | 5 | 0.32 | High | 0 |
| 7. | Daidzein | 254.24 | 4 | 2 | 2.24 | High | 0 |
| 8. | Delphinidin | 338.7 | 7 | 6 | -0.98 | High | 1 |
| 9. | Diosmetin | 300.26 | 6 | 3 | 2.19 | High | 0 |
| 10. | Epiafzelechin | 274.27 | 5 | 4 | 1.17 | High | 0 |
| 11. | Eupatorin | 344.32 | 7 | 2 | 2.53 | High | 0 |
| 12. | Fisetin | 286.24 | 6 | 4 | 1.55 | High | 0 |
| 13. | Formononetin | 268.26 | 4 | 1 | 2.66 | High | 0 |
| 14. | Gallocatechin | 306.27 | 7 | 6 | 0.42 | High | 1 |
| 15. | Hesperidin | 610.56 | 15 | 8 | -0.72 | Low | 3 |
| 16. | Isorhamnetin | 316.26 | 7 | 4 | 1.65 | High | 0 |
| 17. | Kaempferol | 286.24 | 6 | 4 | 1.58 | High | 0 |
| 18. | Luteolin | 286.24 | 6 | 4 | 1.73 | High | 0 |
| 19. | Myricetin | 318.24 | 8 | 6 | 0.79 | Low | 1 |
| 20. | Naringenin | 272.25 | 5 | 3 | 1.84 | High | 0 |
| 21. | Petunidin | 317.27 | 7 | 5 | 0.63 | High | 0 |
| 22. | Pinocembrin | 256.25 | 4 | 2 | 2.26 | High | 0 |
| 23. | Quercetin | 302.24 | 7 | 5 | 1.23 | High | 0 |
| 24. | Rutin | 610.52 | 16 | 10 | -1.29 | Low | 3 |
| 25. | Simvastatin (Control) | 418.57 | 5 | 7 | 4.13 | High | 0 |
| 26. | Sinensetin | 372.37 | 7 | 0 | 3.1 | High | 0 |
| 27. | Taxifolin | 304.25 | 7 | 5 | 0.63 | High | 0 |
| 28. | Theaflavin | 564.49 | 12 | 9 | 1.31 | Low | 3 |
| 29. | Tilianin | 446.4 | 10 | 5 | 0.81 | Low | 0 |

Note: Molecular weight (<500g/mol: satisfactory); Hydrogen bond acceptors (≤10: satisfactory); hydrogen bond donors (≤5: satisfactory); Log P (<5: satisfactory); Violation (≤1: Acceptable).
