# Supplementary material for: Identification of flavonoids from natural products against hyperlipidaemia through computational approaches
Source: PLoS One. 2026 Oct 9;21(10):e0346073. doi: 10.1371/journal.pone.0346073 (PMC13652269; doi:10.1371/journal.pone.0346073)
Supplement: Supplementary file 3 [file pone.0346073.s003.docx]

**Table S2.** ADME Properties data of the following flavonoids and FDA-approved medicine.

|  | Flavonoids | **Absorption** | | | | | | **Distribution** | | | **Metabolism** | | | | | | | | | | | **Excretion** | | |
| --- | --- | --- | --- | --- | --- | --- | --- | --- | --- | --- | --- | --- | --- | --- | --- | --- | --- | --- | --- | --- | --- | --- | --- | --- |
|  |  | Human intestinal absorption (HIA) | Caco-2 permeability (log cm/s) | P-glycoprotein inhibitor | P-glycoprotein substrate | F20% | F30% | (PBB) (%) | (BBB) (cm/s) | Volume distribution (L/kg) | CYP1A2 Substrate | CYP1A2 Inhibitor | CYP2C19 Substrate | CYP2C19  Inhibitor | CYP2C9 Substrate | CYP2C9 Inhibitor | CYP2D6 Substrate | CYP2D6 Inhibitor | CYP3A4  Substrate | CYP3A4 Inhibitor | Half-life time (T1/2) | | Clearance (mL/min/kg) |  |
| 1. | Apigenin | --- | -4.847 | --- | ++ | +++ | +++ | 97.255 | --- | 0.510 | -- | +++ | --- | + | +++ | + | ++ | ++ | -- | ++ | 0.856 | | 7.022 |  |
| 2. | Baicalein | --- | -4.981 | --- | -- | +++ | +++ | 98.992 | --- | 0.436 | -- | +++ | --- | -- | + | + | -- | + | --- | -- | 0.881 | | 4.082 |  |
| 3. | Catechin | --- | -5.971 | --- | --- | +++ | +++ | 92.065 | --- | 0.661 | -- | -- | --- | --- | ++ | -- | - | -- | -- | - | 0.853 | | 17.911 |  |
| 4. | Cyanidin | --- | -5.344 | --- | --- | +++ | +++ | 95.431 | --- | 0.609 | -- | ++ | --- | --- | + | -- | -- | -- | --- | -- | 0.928 | | 15.311 |  |
| 5. | Daidzein | --- | -4.643 | --- | +++ | -- | ++ | 97.079 | --- | 0.495 | -- | +++ | --- | ++ | +++ | - | ++ | +++ | -- | +++ | 0.846 | | 7.802 |  |
| 6. | Delphinidin | --- | -5.826 | --- | --- | +++ | +++ | 93.494 | --- | 0.625 | -- | - | --- | --- | - | -- | -- | --- | --- | -- | 0.944 | | 14.041 |  |
| 7. | Diosmetin | --- | -4.916 | --- | +++ | +++ | +++ | 96.043 | --- | 0.657 | ++ | +++ | --- | -- | +++ | + | ++ | + | -- | ++ | 0.855 | | 7.066 |  |
| 8. | Epiafzelechin | --- | -5.457 | --- | --- | +++ | +++ | 91.971 | --- | 0.936 | -- | - | --- | -- | +++ | -- | ++ | + | -- | - | 0.790 | | 15.780 |  |
| 9. | Eupatorin | --- | -4.836 | +++ | --- | --- | --- | 88.720 | --- | 0.733 | +++ | ++ | -- | - | ++ | ++ | ++ | -- | - | - | 0.707 | | 4.438 |  |
| 10. | Fisetin | --- | -4.987 | --- | --- | +++ | +++ | 97.043 | --- | 0.477 | -- | +++ | --- | --- | + | + | - | + | --- | + | 0.920 | | 8.273 |  |
| 11. | Formononetin | --- | -4.647 | --- | +++ | --- | ++ | 97.256 | -- | 0.468 | ++ | +++ | --- | ++ | +++ | + | +++ | +++ | -- | ++ | 0.547 | | 5.497 |  |
| 12. | Gallocatechin | -- | -6.306 | --- | --- | +++ | +++ | 91.158 | --- | 0.572 | -- | --- | --- | --- | + | -- | -- | --- | -- | -- | 0.870 | | 17.081 |  |
| 13. | Isorhamnetin | --- | -5.056 | --- | --- | --- | +++ | 96.235 | --- | 0.647 | ++ | +++ | --- | -- | ++ | + | - | + | --- | + | 0.922 | | 6.991 |  |
| 14. | Kaempferol | --- | -4.974 | --- | --- | ++ | +++ | 97.861 | --- | 0.522 | -- | +++ | --- | -- | ++ | + | -- | ++ | --- | + | 0.905 | | 6.868 |  |
| 15. | Luteolin | --- | -5.028 | --- | -- | +++ | +++ | 95.436 | --- | 0.533 | -- | +++ | --- | -- | ++ | + | + | + | --- | + | 0.898 | | 8.146 |  |
| 16. | Myricetin | --- | -5.653 | --- | --- | +++ | +++ | 92.766 | --- | 0.633 | -- | ++ | --- | --- | - | + | -- | --- | --- | -- | 0.945 | | 7.716 |  |
| 17. | Naringenin | --- | -4.803 | --- | --- | +++ | +++ | 93.763 | --- | 0.502 | -- | +++ | --- | ++ | +++ | ++ | ++ | ++ | -- | ++ | 0.774 | | 17.388 |  |
| 18. | Petunidin | --- | -5.490 | --- | -- | +++ | +++ | 93.213 | --- | 0.662 | ++ | ++ | --- | --- | + | -- | -- | --- | --- | --- | 0.933 | | 12.517 |  |
| 19. | Pinocembrin | --- | -4.697 | --- | --- | ++ | +++ | 94.129 | -- | 0.495 | -- | +++ | --- | ++ | +++ | ++ | ++ | ++ | -- | + | 0.658 | | 14.249 |  |
| 20. | Quercetin | --- | -5.204 | --- | --- | +++ | +++ | 95.496 | --- | 0.579 | -- | +++ | --- | --- | + | + | -- | - | --- | - | 0.929 | | 8.284 |  |
| 21. | Simvastatin (Control) | --- | -4.732 | +++ | --- | +++ | +++ | 97.149 | + | 1.163 | --- | --- | ++ | --- | --- | - | - | --- | ++ | ++ | 0.104 | | 15.125 |  |
| 22. | Sinensetin | --- | -4.563 | +++ | --- | --- | --- | 71.403 | -- | 0.813 | +++ | - | ++ | + | +++ | + | +++ | --- | ++ | + | 0.468 | | 5.809 |  |
| 23. | Taxifolin | --- | -6.060 | --- | --- | ++ | +++ | 93.228 | --- | 0.556 | --- | - | --- | --- | ++ | - | -- | - | -- | ++ | 0.759 | | 12.286 |  |
| 24. | Tilianin | ++ | -5.607 | --- | ++ | --- | +++ | 78..814 | -- | 0.962 | -- | --- | -- | --- | ++ | --- | + | --- | --- | --- | 0.245 | | 2.334 |  |
