# Supplementary material for: Identification of flavonoids from natural products against hyperlipidaemia through computational approaches
Source: PLoS One. 2026 Oct 9;21(10):e0346073. doi: 10.1371/journal.pone.0346073 (PMC13652269; doi:10.1371/journal.pone.0346073)
Supplement: Supplementary file 4 [file pone.0346073.s004.docx]

**Table S3.** Toxicity prediction of the flavonoids and Simvastatin by using FDA-approved medicine.

|  | **Properties** | | | | | | | | |
| --- | --- | --- | --- | --- | --- | --- | --- | --- | --- |
|  | **Flavonoids** | **hERG Blockers** | **Human Hepatotoxicity (H-HT)** | **Ames Toxicity** | **Rat Oral Acute Toxicity** | **Respiratory Toxicity** | **Carcinogenicity** | **SkinSen** | **Drug-Induced Liver Injury (DILI)** |
| 1. | Apigenin | --- | --- | - | --- | -- | -- | +++ | ++ |
| 2. | Baicalein | --- | --- | - | -- | - | - | +++ | +++ |
| 3. | Catechin | --- | --- | + | - | -- | --- | +++ | -- |
| 4. | Cyanidin | --- | -- | + | --- | -- | --- | +++ | +++ |
| 5. | Daidzein | --- | --- | --- | - | -- | + | ++ | + |
| 6. | Delphinidin | -- | -- | - | --- | -- | --- | +++ | +++ |
| 7. | Diosmetin | --- | --- | + | --- | - | --- | +++ | ++ |
| 8. | Epiafzelechin | --- | --- | -- | - | -- | -- | +++ | --- |
| 9. | Eupatorin | -- | --- | - | --- | - | --- | ++ | ++ |
| 10. | Fisetin | --- | -- | ++ | -- | --- | -- | +++ | +++ |
| 11. | Formononetin | -- | --- | -- | -- | - | - | ++ | + |
| 12. | Gallocatechin | --- | --- | - | -- | --- | --- | +++ | -- |
| 13. | Isorhamnetin | --- | --- | + | --- | -- | --- | ++ | +++ |
| 14. | Kaempferol | --- | --- | + | -- | --- | --- | ++ | +++ |
| 15. | Luteolin | --- | --- | + | --- | -- | --- | +++ | +++ |
| 16. | Myricetin | -- | --- | - | --- | --- | --- | +++ | +++ |
| 17. | Naringenin | --- | --- | - | + | - | + | +++ | ++ |
| 18. | Petunidin | -- | -- | - | --- | -- | --- | +++ | +++ |
| 19. | Pinocembrin | --- | -- | - | + | + | + | ++ | ++ |
| 20. | Quercetin | --- | --- | + | --- | --- | --- | +++ | +++ |
| 21. | Simvastatin (Control) | + | +++ | --- | - | + | ++ | +++ | --- |
| 22. | Sinensetin | -- | --- | -- | -- | -- | --- | - | ++ |
| 23. | Taxifolin | --- | -- | + | -- | -- | --- | +++ | +++ |
| 24. | Tilianin | --- | --- | ++ | --- | --- | ++ | --- | +++ |

*For the classification endpoints, the prediction probability values are transformed into six symbols: 0-0.1 (---), 0.1-0.3 (--), 0.3-0.5 (-), 0.5-0.7 (+), 0.7-0.9(++), and 0.9-1.0 (+++).
