# Supplementary material for: Identification of flavonoids from natural products against hyperlipidaemia through computational approaches
Source: PLoS One. 2026 Oct 9;21(10):e0346073. doi: 10.1371/journal.pone.0346073 (PMC13652269; doi:10.1371/journal.pone.0346073)
Supplement: Supplementary file 5 [file pone.0346073.s005.docx]

**Table S4.** Toxicity Prediction of the flavonoids and FDA-approved medicine using ProTox II.

|  |  | **LD50**  **(mg/kg)** | **Toxicity** | | | | | | | | | | |  |
| --- | --- | --- | --- | --- | --- | --- | --- | --- | --- | --- | --- | --- | --- | --- |
|  | **Flavonoids** |  | **Hepatoxicity** | | **Carcinogenicity** | | **Immunotoxicity** | | **Mutagenicity** | | **Cytotoxicity** | | **Toxicity Class** | **Prediction Accuracy** |
|  |  |  | **Prediction** | **Probability** | **Prediction** | **Probability** | **Prediction** | **Probability** | **Prediction** | **Probability** | **Prediction** | **Probability** |  |  |
| 1. | Apigenin | 2500 | Inactive | 0.68 | Inactive | 0.62 | Inactive | 0.99 | Inactive | 0.57 | Inactive | 0.87 | 5 | 70.97 |
| 2. | Baicalein | 3919 | Inactive | 0.69 | Active | 0.68 | Inactive | 0.99 | Active | 0.51 | Inactive | 0.99 | 5 | 70.97 |
| 3. | Catechin | 10000 | Inactive | 0.72 | Inactive | 0.51 | Inactive | 0.96 | Inactive | 0.55 | Inactive | 0.84 | 6 | 100 |
| 4. | Cyanidin | 5000 | Inactive | 0.72 | Active | 0.61 | Inactive | 0.93 | Inactive | 0.94 | Inactive | 0.94 | 5 | 69.26 |
| 5. | Daidzein | 2430 | Inactive | 0.71 | Inactive | 0.58 | Inactive | 0.92 | Inactive | 0.85 | Inactive | 0.89 | 5 | 72.9 |
| 6. | Delphinidin | 5000 | Inactive | 0.71 | Inactive | 0.71 | Inactive | 0.86 | Inactive | 0.76 | Inactive | 0.85 | 5 | 69.26 |
| 7. | Diosmetin | 3919 | Inactive | 0.72 | Inactive | 0.68 | Inactive | 0.61 | Inactive | 0.94 | Inactive | 0.95 | 5 | 70.97 |
| 8. | Epiafzelechin | 2500 | Inactive | 0.71 | Inactive | 0.70 | Inactive | 0.98 | Inactive | 0.56 | Inactive | 0.84 | 5 | 70.97 |
| 9. | Eupatorin | 4000 | Inactive | 0.70 | Inactive | 0.63 | Active | 0.96 | Inactive | 0.77 | Inactive | 0.66 | 5 | 69.26 |
| 10. | Fisetin | 159 | Inactive | 0.7 | Active | 0.71 | Inactive | 0.51 | Inactive | 0.53 | Inactive | 0.98 | 3 | 72.9 |
| 11. | Formononetin | 2500 | Inactive | 0.73 | Active | 0.50 | Active | 0.54 | Inactive | 0.74 | Inactive | 0.88 | 5 | 70.97 |
| 12. | Gallocatechin | 10000 | Inactive | 0.72 | Inactive | 0.51 | Inactive | 0.92 | Inactive | 0.55 | Inactive | 0.84 | 6 | 100 |
| 13. | Isorhamnetin | 5000 | Inactive | 0.72 | Inactive | 0.68 | Active | 0.58 | Inactive | 0.94 | Inactive | 0.95 | 5 | 70.97 |
| 14. | Kaempferol | 3919 | Inactive | 0.68 | Inactive | 0.72 | Inactive | 0.96 | Inactive | 0.52 | Inactive | 0.98 | 5 | 70.97 |
| 15. | Luteolin | 3919 | Inactive | 0.69 | Active | 0.68 | Inactive | 0.97 | Active | 0.51 | Inactive | 0.99 | 5 | 70.97 |
| 16. | Myricetin | 159 | Inactive | 0.69 | Active | 0.68 | Inactive | 0.86 | Active | 0.51 | Inactive | 0.99 | 3 | 100 |
| 17. | Naringenin | 2000 | Inactive | 0.67 | Inactive | 0.62 | Inactive | 0.88 | Inactive | 0.83 | Active | 0.59 | 4 | 69.26 |
| 18. | Petunidin | 5000 | Inactive | 0.71 | Active | 0.52 | Active | 0.72 | Inactive | 0.63 | Inactive | 0.91 | 5 | 69.26 |
| 19. | Pinocembrin | 2000 | Inactive | 0.67 | Inactive | 0.62 | Inactive | 0.93 | Inactive | 0.83 | Active | 0.59 | 4 | 69.26 |
| 20. | Quercetin | 159 | Inactive | 0.69 | Active | 0.68 | Inactive | 0.87 | Active | 0.51 | Inactive | 0.99 | 3 | 100 |
| 21. | Simvastatin (Control) | 1000 | Inactive | 0.89 | Active | 0.64 | Active | 0.99 | Inactive | 0.87 | Inactive | 0.82 | 4 | 100 |
| 22. | Sinensetin | 5000 | Inactive | 0.69 | Inactive | 0.53 | Active | 0.91 | Inactive | 0.69 | Inactive | 0.82 | 5 | 69.26 |
| 23. | Taxifolin | 2000 | Inactive | 0.69 | Active | 0.68 | Inactive | 0.76 | Active | 0.51 | Inactive | 0.99 | 4 | 100 |
| 24. | Tilianin | 5000 | Inactive | 0.84 | Inactive | 0.90 | Inactive | 0.58 | Active | 0.58 | Inactive | 0.59 | 5 | 69.26 |

Using Pro Tox II, LD50 values and toxicological class were calculated. All compounds from series 2 have an LD50 value of 5000 mg/kg. All compounds are class 3 (toxic if swallowed: 50 < LD50 ≤ 300, class 4 (harmful if swallowed: 300 < LD50 ≤ 2000) and class 5 (may be harmful if swallowed: 2000 < LD50 ≤ 5000).
