# Supplementary material for: Identification of flavonoids from natural products against hyperlipidaemia through computational approaches
Source: PLoS One. 2026 Oct 9;21(10):e0346073. doi: 10.1371/journal.pone.0346073 (PMC13652269; doi:10.1371/journal.pone.0346073)
Supplement: Supplementary file 6 [file pone.0346073.s006.docx]

**Table S5.** Validation results of refinement PCSK9

| Programme | Calculated Values | |
| --- | --- | --- |
| RMSD | 0.075 Å | |
| ERRAT | 91.6522% | |
| VERIFY3D | 67.85% | |
| Stereochemical Parameter  (Ramachandran Plot) | Calculated Values (%) | |
|  | PCSK9 | |
| Residue in most favored regions | 478 | 90.5 |
| Residue in the additionally allowed zoned | 46 | 8.7 |
| Residue in the generously region | 2 | 0.4 |
| Residue in disallowed regions | 2 | 0.4 |
