# Supplementary material for: Identification of flavonoids from natural products against hyperlipidaemia through computational approaches
Source: PLoS One. 2026 Oct 9;21(10):e0346073. doi: 10.1371/journal.pone.0346073 (PMC13652269; doi:10.1371/journal.pone.0346073)
Supplement: Supplementary file 7 [file pone.0346073.s007.docx]

**Table S6.** Statistical analysis of RMSD backbone in triplicate data for all MD simulation.

| **Ligands** | **Parameter** | **1^st^ Run** | **2^nd^ Run** | **3^rd^ run** | **Average (Mean)** | **Standard Deviation** |
| --- | --- | --- | --- | --- | --- | --- |
| Simvastatin (control) | RMSD Backbone (nm) | 0.2168 | 0.1942 | 0.2348 | 0.215 | 0.017 |
| Diosmetin | RMSD Backbone (nm) | 0.2537 | 0.2641 | 0.2229 | 0.247 | 0.017 |
| Eupatorin | RMSD Backbone (nm) | 0.2228 | 0.2276 | 0.2623 | 0.238 | 0.017 |
| Isorhamnetin | RMSD Backbone (nm) | 0.247 | 0.2081 | 0.2907 | 0.249 | 0.034 |
| Myricetin | RMSD Backbone (nm) | 0.2261 | 0.2339 | 0.2533 | 0.238 | 0.011 |
| Petunidin | RMSD Backbone (nm) | 0.2253 | 0.2407 | 0.2124 | 0.226 | 0.012 |
| Sinensetin | RMSD Backbone (nm) | 0.2611 | 0.217 | 0.2936 | 0.257 | 0.031 |
| Tilianin | RMSD Backbone (nm) | 0.2224 | 0.1979 | 0.2541 | 0.225 | 0.023 |
