# Supplementary material for: Identification of flavonoids from natural products against hyperlipidaemia through computational approaches
Source: PLoS One. 2026 Oct 9;21(10):e0346073. doi: 10.1371/journal.pone.0346073 (PMC13652269; doi:10.1371/journal.pone.0346073)
Supplement: Supplementary file 8 [file pone.0346073.s008.docx]

**Table S7.** Decomposition of MM/PBSA binding free energy components for PCSK9-ligand complexes calculated from the equilibrated MD simulations.

| **Energy** | **Energy Values kcal/mol** | | | | | | | | | | | | | | | |
| --- | --- | --- | --- | --- | --- | --- | --- | --- | --- | --- | --- | --- | --- | --- | --- | --- |
|  | **Simvastatin (Control)** | | **Diosmetin** | | **Eupatorin** | | **Isorhamnetin** | | **Myricetin** | | **Petunidin** | | **Sinensetin** | | **Tilianin** | |
|  | **GB** | **PB** | **GB** | **PB** | **GB** | **PB** | **GB** | **PB** | **GB** | **PB** | **GB** | **PB** | **GB** | **PB** | **GB** | **PB** |
| **VDWAALS** | -46.83 | | -36.87 | | -33.68 | | -40.24 | | -29.34 | | -39.10 | | -47.04 | | -53.32 | |
| **EEL** | -16.47 | | -4.87 | | -12.73 | | -29.29 | | -58.41 | | -102.05 | | -22.33 | | -63.00 | |
| **EGB/EPB** | 28.28 | 37.71 | 26.95 | 45.62 | 27.29 | 31.55 | 42.42 | 55.52 | 57.42 | 63.33 | 118.33 | 126.77 | 41.43 | 54.86 | 79.51 | 92.24 |
| **ESURF** | -5.59 | N/A | -4.80 | N/A | -4.43 | N/A | -5.11 | N/A | -5.02 | N/A | -4.95 | N/A | -5.80 | N/A | -7.09 | N/A |
| **ENPOLAR** | N/A | -4.82 | N/A | -3.76 | N/A | -3.78 | N/A | -3.84 | N/A | -3.67 | N/A | -3.86 | N/A | -4.57 | N/A | -5.09 |
| **DELTA G Gas** | -63.30 | -63.30 | -41.74 | -41.74 | -46.41 | -46.41 | -69.53 | -69.53 | -87.75 | -87.75 | -141.15 | -141.15 | -69.38 | -69.38 | -116.32 | -116.32 |
| **DELTA G**  **solv** | 22.69 | 32.88 | 22.15 | 26.67 | 22.86 | 27.77 | 37.31 | 51.68 | 52.40 | 59.66 | 113.38 | 122.91 | 35.63 | 50.29 | 72.42 | 87.14 |
| **Total** | -40.61 | -30.41 | -19.59 | -15.07 | -23.55 | -18.64 | -32.23 | -17.86 | -35.35 | -28.09 | -27.77 | -18.24 | -33.75 | -19.09 | -43.90 | -29.18 |
