# Supplementary material for: Identification of flavonoids from natural products against hyperlipidaemia through computational approaches
Source: PLoS One. 2026 Oct 9;21(10):e0346073. doi: 10.1371/journal.pone.0346073 (PMC13652269; doi:10.1371/journal.pone.0346073)
Supplement: Supplementary file 9 [file pone.0346073.s010.docx]

**Reviewer 1**

In this manuscript titled "Identification of Flavonoids from Natural products against Hyperlipidaemia through Computational Approaches", authors have evaluated pharmacological potential of flavonoids to identify strong inhibitors for target receptor, Proprotein Convertase Subtilisin/Kexin Type (PCSK9).

The results revealed that the flavonoids evaluated, Tilianin, Myricetin, and Sinensetin demonstrate the most favourable interaction profiles.
These finding suggest that the selected flavonoids have strong potential as promising anti-hyperlipidaemic candidates targeting PCSK9.

Although the work seems interesting, a thorough revision is required before its consideration:

| **Section** | **Comment Reviewer** | **Statement** |
| --- | --- | --- |
| 1. | Abstract seems good with brief summary of the performed work. | Thank you for the generous comment on this article. |
| 2. | Introduction: Seems weak with lack of research gaps. Authors are strongly encouraged to go through the following relevant research articles and include them accordingly. https://doi.org/10.1016/j.ejphar.2020.173690 https://doi.org/10.1016/j.phymed.2023.155258 https://doi.org/10.1016/j.intimp.2024.112178 10.1038/s41401-024-01305-9 10.1021/acs.analchem.3c04746 https://doi.org/10.1016/j.molstruc.2024.139269 https://doi.org/10.1016/j.heliyon.2024.e37811 10.1039/D2FO02298B <https://doi.org/10.26599/FSHW.2024.9250007> | - We appreciate the reviewer’s comment regarding the lack of gaps. - We have added the relevant research articles in the introductions (Page 2, Line 32), (Page 3, Line 31). - We also rewrite and improve the introduction to address research gaps more clearly (Page 2-4). |
| 3. | Authors should provide more information on section 2.2.2 Refinement of PCSK9. | - We have added more information on this section for refinement of PCSK9 on (Page 6, Line 26-34), (Page 7, 1-4) . |
| 4. | Can the authors detail on the chemical bonding involved in Structural Flexibility? | - We have explained more details regarding chemical bonding involved in structural flexibility in Page 18 (Table 4). However, on the specific residues involved to help in flexibility can be find in the next section which is 3.4.2.1 Hyrdrogen bond analysis. |
| 5. | Conclusion section can involve numerical results (like abstract). | - We have included numerical results like abstract in conclusion on Page 24 (Line 18-20). |

**Reviewer 2**

| **Section** | **Comment Reviewer** | **Statement** |
| --- | --- | --- |
| 1. | The manuscript requires a thorough linguistic overhaul. There are numerous grammatical errors, typos, and poor phrasing choices throughout the mansucript that obscure the scientific content. | - We thank the reviewer for this valuable suggestion. The manuscript has been thoroughly revised to improve its overall linguistic quality. Grammatical errors, spelling mistakes, punctuation, sentence structure, and word choice have been carefully corrected throughout the manuscript. In addition, several sections were rephrased to improve clarity, readability, and scientific precision while preserving the original meaning. We believe these revisions have substantially enhanced the presentation of the study and made the scientific content clearer for readers. |
|  | There is a noticeable gap in the biological rationale connecting the chosen plant extracts with the molecular targets. While targeting the PCSK9 pathway is a sound approach, the introduction fails to establish a clear, mechanistic link between flavonoids and this specific protein. | - The introduction has been revised to provide a clearer biological rationale linking flavonoids to PCSK9 as the molecular target. Specifically, we expanded the discussion describing the lipid-lowering mechanisms of flavonoids through modulation of the SREBP-2/HNF-1α/PCSK9 pathway and clarified that the polyphenolic scaffold of flavonoids enables favorable non-covalent interactions with proteins, supporting their potential to directly interact with PCSK9 (Page 3, Lines 13-31). We also strengthened the research gap by emphasizing that the direct molecular interactions between flavonoids and PCSK9 remain insufficiently characterized, thereby providing the rationale for the present computational investigation (Page 4, Lines 8-17). |
|  | A critical accuracy issue arises regarding Talinum triangulare: the text claims its flavonoid extract (TTFE) addresses dyslipidemia by "enhancing insulin secretion through antioxidant mechanisms." However, established pharmacological literature (e.g., Oluba et al.) shows that TTFE’s lipid-lowering effects are primarily driven by the direct inhibition of the HMG-CoA reductase enzyme. The authors must correct this claim and provide the accurate bibliographical reference. | - The revised text now states that TTFE ameliorates dyslipidemia primarily through inhibition of HMG-CoA reductase, thereby reducing hepatic cholesterol synthesis, in accordance with the findings of Oluba et al. In addition, the effects of TTFE on plasma insulin secretion, α-amylase inhibition, and oxidative stress are now presented separately as its antidiabetic activities rather than as the mechanism underlying its lipid-lowering effect. The appropriate reference has also been included (Page 3, Lines 25-31). |
|  | Furthermore, the manuscript needs to explain why flavonoids are viable candidates for this study. The authors should explicitly note that various flavonoid families are well-documented modulators of REBP-2 and HNF-1A, the key upstream transcription factors that dictate PCSK9 gene expression. This addition is vital to justify why these natural compounds are being screened against this specific target. | - We added a discussion describing how several flavonoid subclasses modulate the upstream transcription factors SREBP-2 and HNF-1α, resulting in reduced PCSK9 expression, enhanced LDLR expression, and improved hepatic LDL-C clearance. We further explained that the structural characteristics of flavonoids support their potential to directly interact with PCSK9, thereby strengthening the rationale for screening these natural compounds against this molecular target (Page 3, Lines 16-24). |
|  | The Materials and Methods section currently lacks the depth required for reproducibility. To ensure the robustness of the in silico workflow, several technical protocols must be clarified. - First, the exact procedure for preparing and cleaning the initial coordinates from the Protein Data Bank (PDB) must be stated. It is necessary to clarify whether crystallographic water molecules, non-essential ions, co-crystals, or endogenous ligands were stripped from the structure, and whether missing loops or residues were reconstructed using homology modeling or specialized software packages. - Second, the system relaxation phase requires a more rigorous description. The manuscript should report the precise simulation times allocated for both the thermal equilibration under the canonical ensemble (NVT) and the pressure equilibration under the isothermal-isobaric ensemble (NPT). The specific thermostat and barostat algorithms used during these stages must also be named. | - Specifically, we have clarified the preprocessing steps applied to the crystal structure obtained from the Protein Data Bank, including the removal of non-essential crystallographic components (Page 6, Line 7-20), reconstruction of missing residues using the SWISS-MODEL server, and subsequent preparation of the refined protein structure for molecular docking. These additional details improve the reproducibility of the computational workflow (Page 6, Lines 21-24). - We have included the duration of the equilibration stages (100 ps for both the NVT and NPT ensembles), the simulation conditions (310 K and 1 bar), and the coupling algorithms used during system equilibration, namely the velocity-rescale (V-rescale) thermostat for temperature coupling and the Berendsen barostat for pressure coupling. These additions improve the reproducibility of the molecular dynamics simulation workflow (Page 8, Lines 9-14). |
|  | Finally, while the binding free energy calculations are noted as being performed via MM-PBSA over the stable 70–100 ns trajectory window, essential parameters are missing. The authors must specify the exact number of snapshots extracted for this analysis, disclose the precise values assigned to the solute and solvent dielectric constants, and break down the specific energetic contributions (such as van der Waals, electrostatic, polar, and non-polar solvation terms) included in the final estimations. | - We have clarified that binding free energy calculations were performed using the equilibrated 70–100 ns trajectory window, from which 31 trajectory snapshots were extracted for analysis. We have also specified that the internal (solute) and external (solvent) dielectric constants were set to the default values of 1.0 and 80.0, respectively, as implemented in gmx_MMPBSA v1.6.3. Furthermore, we have expanded the description of the MM/PBSA calculations to explicitly state that the total binding free energy (ΔG_bind) comprises van der Waals, electrostatic, polar solvation, and non-polar solvation energy contributions. These revisions improve the transparency and reproducibility of the computational protocol (Page 8, Line 25-29). |

**Reviewer 3**

| **Section** | **Comment Reviewer** | **Statement** |
| --- | --- | --- |
| 1. | Give a list of abbreviations for all abreviated terms found in the manuscript. Revise the total binding free energy (MMPBSA) regarding some flavonoids with PCSK9, like sinensetin (−23.48 kcal/mol) and Eupatorin (−20.97 kcal/mol) that must align with the corresponding values in Fig.7. | - The abbreviations were defined at their first appearance in the text. In addition, we carefully reviewed the reported MM/PBSA binding free energy values and compared them with the data presented in Figure 7. The manuscript has been revised to ensure that the reported total binding free energy values are fully consistent with those shown in Figure 7. We have carefully verified all reported MM/PBSA values to maintain consistency throughout the manuscript (Page 20, Line 5-10) |
|  | What docking reference is used in this table, and how does the binding affinity score (kcal/mol) for each flavonoid compare to it? Mention the RMSD of the docking reference to validate your docking process. | - Thank you for this valuable comment. We have revised the manuscript to clearly indicate that simvastatin was used as the reference compound for comparison of the docking binding affinities of the flavonoids. The corresponding text has been updated to compare the binding affinity of each flavonoid with that of simvastatin. - Regarding docking validation, we acknowledge the reviewer's suggestion. However, the selected receptor structure (PDB ID: 5OCA) does not contain a co-crystallized small-molecule ligand that would permit a conventional redocking validation and RMSD calculation. The structure comprises PCSK9 in complex with a Fab antibody and a heparan sulfate (dextran sulfate) oligosaccharide rather than a drug-like ligand. Consequently, an RMSD-based redocking validation was not applicable in this study. To strengthen the reliability of the docking predictions, we employed complementary validation approaches, including 100 ns triplicate molecular dynamics simulations, MM/PBSA binding free energy calculations, and experimental in vitro PCSK9 inhibition assays. |
|  | Provide further information regarding eupatorin's primary pharmacological actions in the introduction, along with any prior research on the drug's effects on cardiovascular disease or other pharmacological functions, all of which should be supported by references that support your hypothesis. | - We have revised the manuscript to include additional discussion of Eupatorin, highlighting its reported antioxidant, anti-inflammatory, vasodilatory, and hepatoprotective properties, as well as its previously reported cardiovascular benefits through nitric oxide-mediated vasorelaxation. We also clarified that, although Eupatorin has demonstrated these pharmacological activities, its direct interaction with PCSK9 has not previously been investigated. Appropriate references have been incorporated to strengthen the rationale for evaluating Eupatorin as one of the flavonoids screened in this study (Page 23, Line 9-12) |
|  | According to the author, Eupatorin essentially interacts with the catalytic domain of PCSK9 through allosteric modulation, inhibiting PCSK9 without directly preventing LDLR binding. Provide a more detailed explanation of allosteric modulation. What is the evidence for this allosteric modulatory effect? A supporting figure or graph illustrating this type of interaction would be useful. Find the primary structural characteristics of Eupatorin that support its allosteric modulatory activity. | - We have revised the discussion to clarify that the proposed allosteric mechanism is a hypothesis derived from the computational findings rather than a confirmed mechanism. We further included a discussion of the polymethoxyflavone structure of Eupatorin, whose methoxy substituents and hydrophobic aromatic scaffold may contribute to its preferential interaction with the catalytic pocket. Supporting references have been added to substantiate this interpretation (Page 22, Line 32-35) (Page 23, Line 1-11). - Regarding the requested illustration, the interaction pattern of Eupatorin is already presented in Figure 6, which depicts the representative two-dimensional protein-ligand interactions obtained from the molecular dynamics simulations. This figure illustrates the catalytic-domain residues involved in Eupatorin binding catalytic domain and was considered sufficient to support the discussion without introducing an additional figure (Page 19) (Figure 6 (d)). |

**Reviewer 4**

| **Section** | **Comment Reviewer** | **Statement** |
| --- | --- | --- |
|  | The manuscript presents an interesting computational screening of flavonoids against PCSK9, but it requires substantial improvement in scientific rigor, consistency, and presentation before it can be considered suitable for publication. The intorduction of the article is not up-to-date, author requests will add the advanced and computational literature in introduction section. For in sililico and computational updapation, authors can follow the following articles: 1)https://doi.org/10.1016/j.molstruc.2021.131915 2) https://link.springer.com/article/10.1186/s43088-021-00117-8 3)https://doi.org/10.33945/SAMI/AJCA.2019.4.5 4)http://doi.org/10.25135/acg.oc.122.2203.2397 5) https://doi.org/10.33945/SAMI/AJCA.2019.2.190202 6) http://dx.doi.org/10.4067/S0717-97072021000205206 7)https://doi.org/10.1016/j.heliyon.2021.e07509 8)https://doi.org/10.3390/molecules27123694 9)https://doi.org/10.1016/j.molstruc.2021.131087 10)https://dergipark.org.tr/en/pub/tcandtc/article/481878 11)<http://dx.doi.org/10.4067/S0717-97072022000305623> | - The introduction has been updated to include recent advances in computational drug discovery and structure-based virtual screening. We incorporated the recommended literature where appropriate to highlight the growing application of integrated computational approaches, including molecular docking, molecular dynamics simulations, binding free energy calculations, and ADMET prediction, in the identification and optimization of bioactive natural products (Page 4, Line 1-7). |
|  | Clarify the study design and workflow, because the manuscript alternates between 28, 29, and 24 compounds and this inconsistency must be corrected throughout the text, figures, and tables. Provide the flavonoid numbering in Table 1. | - We have thoroughly revised the manuscript to ensure consistency in the reporting of compound numbers throughout the text, figures, and tables. The study initially included 29 compounds, comprising 28 naturally occurring flavonoids and one FDA-approved drug (Simvastatin) used as the reference control (Refer Figure 2). Following drug-likeness and pharmacokinetic screening, 24 flavonoids satisfied the selection criteria and were subjected to molecular docking. Based on the docking results, seven flavonoids, together with Simvastatin, were selected for molecular dynamics simulations, MM/PBSA analysis, in vitro PCSK9–LDLR inhibition assays, and in vivo zebrafish toxicity evaluation. In addition, sequential numbering has been added to all flavonoids in Table 1 to improve clarity and facilitate cross-referencing throughout the manuscript (Page 5 & 6). |
|  | Recheck all numerical results and ensure they are internally consistent, especially docking scores, MD descriptors, and MM-PBSA values, since several values and ligand rankings appear to be presented differently in different sections. | - We have carefully rechecked all numerical data presented throughout the manuscript, including docking scores, molecular dynamics descriptors (RMSD, RMSF, SASA, radius of gyration, hydrogen bonds), and MM/PBSA binding free energies. All values, ligand rankings, and corresponding interpretations have been verified against the original datasets and revised where necessary to ensure complete consistency across the Abstract, Results, Discussion, figures, tables, and Supplementary Information. |
|  | Improve the methodological description so the refinement, docking, and MD simulation steps can be reproduced precisely, including protein preparation, ligand preparation, grid settings, simulation conditions, replicates, and analysis parameters. | - We have revised the Materials and Methods section to improve the reproducibility of the computational workflow. Additional methodological details have been included for protein and ligand preparation, receptor refinement, molecular docking, molecular dynamics simulations, and trajectory analyses. Specifically, we have clarified the receptor and ligand preparation procedures, docking grid dimensions and coordinates, simulation parameters (force field, water model, equilibration conditions, production run, and triplicate simulations), and the analysis protocols used to evaluate structural stability, flexibility, hydrogen bond occupancy, pocket volume, and MM/PBSA binding free energies. These revisions provide a more comprehensive and reproducible description of the computational methodology) (Page 8, Line 1-34). |
|  | Strengthen the rationale for choosing PDB ID 5OCA and explain more clearly why this structure is appropriate for PCSK9-targeted inhibitor screening. | - The Materials and Methods section has been revised to provide a clearer justification for selecting PDB ID 5OCA as the receptor model. Specifically, we now state that this human PCSK9 crystal structure was chosen because of its high crystallographic resolution (2.30 Å), which provides reliable atomic coordinates and well-defined binding pockets for molecular docking. In addition, we clarify that the structure preserves the catalytic and C-terminal V-domains of PCSK9, which are directly involved in ligand recognition and are therefore suitable for identifying potential small-molecule binding sites. These additions better explain the suitability of PDB ID 5OCA for structure-based virtual screening and molecular dynamics simulations (Page 6, Line 8-13) |
|  | Provide a more critical interpretation of the computational findings, because favorable docking or MM-PBSA values alone are not sufficient to claim therapeutic promise without experimental validation. | - The discussion has been revised to provide a more balanced interpretation of the computational findings. We now explicitly acknowledge that molecular docking, molecular dynamics simulations, and MM/PBSA analyses are predictive computational approaches that evaluate binding modes, complex stability, and estimated binding free energies but do not directly demonstrate biological inhibitory activity or therapeutic efficacy (Page 23, Line 31-36), (Page 24, Line 1-5) |
|  | Check reference formatting carefully, since several citations appear incomplete or inconsistently styled.  Rewrite the conclusions regarding the objectives of introduction. | - All in-text citations and reference entries have been carefully reviewed and revised to ensure consistency with the journal's reference formatting guidelines. Inconsistent citation styles have been corrected, incomplete references have been completed where necessary, and all references have been checked for accuracy and uniformity throughout the manuscript. - The revised conclusion now summarizes the principal findings according to each objective, including the identification of flavonoids with favourable drug-likeness and pharmacokinetic properties, the evaluation of their interactions with PCSK9 through molecular docking and molecular dynamics simulations, and the identification of the most promising lead compounds. The conclusion also emphasizes the predictive nature of the computational findings and the need for subsequent experimental validation (Page 25, Line 7-24). |
