# Supplementary material for: Identification of flavonoids from natural products against hyperlipidaemia through computational approaches
Source: PLoS One. 2026 Oct 9;21(10):e0346073. doi: 10.1371/journal.pone.0346073 (PMC13652269; doi:10.1371/journal.pone.0346073)
Supplement: Peer review history

# Peer review: Decision Letter 0

**DOI:** 10.1371/journal.pone.0346073.r001

**Authors:** Jorddy Neves Cruz

29 Jun 2026

PONE-D-26-12566

Identification of Flavonoids from Natural products against Hyperlipidaemia through Computational Approaches

PLOS One

Dear Dr. Ismail,

Thank you for submitting your manuscript to PLOS ONE. After careful consideration, we feel that it has merit but does not fully meet PLOS ONE’s publication criteria as it currently stands. Therefore, we invite you to submit a revised version of the manuscript that addresses the points raised during the review process.

==============================

Please submit your revised manuscript by Aug 14 2026 11:59PM. If you will need more time than this to complete your revisions, please reply to this message or contact the journal office at plosone@plos.org. When you're ready to submit your revision, log on to https://www.editorialmanager.com/pone/ and select the 'Submissions Needing Revision' folder to locate your manuscript file.

Please include the following items when submitting your revised manuscript:

- A letter that responds to each point raised by the academic editor and reviewer(s). You should upload this letter as a separate file labeled 'Response to Reviewers'.
- A marked-up copy of your manuscript that highlights changes made to the original version. You should upload this as a separate file labeled 'Revised Manuscript with Track Changes'.
- An unmarked version of your revised paper without tracked changes. You should upload this as a separate file labeled 'Manuscript'.

If you would like to make changes to your financial disclosure, please include your updated statement in your cover letter. Guidelines for resubmitting your figure files are available below the reviewer comments at the end of this letter.

If applicable, we recommend that you deposit your laboratory protocols in protocols.io to enhance the reproducibility of your results. Protocols.io assigns your protocol its own identifier (DOI) so that it can be cited independently in the future. For instructions see: https://journals.plos.org/plosone/s/submission-guidelines#loc-laboratory-protocols. Additionally, PLOS ONE offers an option for publishing peer-reviewed Lab Protocol articles, which describe protocols hosted on protocols.io. Read more information on sharing protocols at https://plos.org/protocols?utm_medium=editorial-email&utm_source=authorletters&utm_campaign=protocols.

As the corresponding author, your ORCID iD is verified in the submission system and will appear in the published article. PLOS supports the use of ORCID, and we encourage all coauthors to register for an ORCID iD and use it as well. Please encourage your coauthors to verify their ORCID iD within the submission system before final acceptance, as unverified ORCID iDs will not appear in the published article. Only the individual author can complete the verification step; PLOS staff cannot verify ORCID iDs on behalf of authors.

We look forward to receiving your revised manuscript.

Kind regards,

Jorddy Neves Cruz

Academic Editor

PLOS One

Journal Requirements:

When submitting your revision, we need you to address these additional requirements.

1. Please ensure that your manuscript meets PLOS ONE's style requirements, including those for file naming. The PLOS ONE style templates can be found at

https://journals.plos.org/plosone/s/file?id=wjVg/PLOSOne_formatting_sample_main_body.pdf and

https://journals.plos.org/plosone/s/file?id=ba62/PLOSOne_formatting_sample_title_authors_affiliations.pdf

2. In your Methods section, please provide additional information regarding the permits you obtained for the work. Please ensure you have included the full name of the authority that approved the field site access and, if no permits were required, a brief statement explaining why.

3.  PLOS requires an ORCID iD for the corresponding author in Editorial Manager on papers submitted after December 6th, 2016. Please ensure that you have an ORCID iD and that it is validated in Editorial Manager. To do this, go to ‘Update my Information’ (in the upper left-hand corner of the main menu), and click on the Fetch/Validate link next to the ORCID field. This will take you to the ORCID site and allow you to create a new iD or authenticate a pre-existing iD in Editorial Manager.

4. Thank you for stating the following financial disclosure:

"The publication of this manuscript was supported by School of Graduate Studies (SGS), Management and Science University, SG-018-022020-SGS."

Please state what role the funders took in the study.  If the funders had no role, please state: "The funders had no role in study design, data collection and analysis, decision to publish, or preparation of the manuscript."

If this statement is not correct you must amend it as needed.

Please include this amended Role of Funder statement in your cover letter; we will change the online submission form on your behalf.

5. We note that your Data Availability Statement is currently as follows: "

All relevant data are within the manuscript and its Supporting Information files."

Please confirm at this time whether or not your submission contains all raw data required to replicate the results of your study. Authors must share the “minimal data set” for their submission. PLOS defines the minimal data set to consist of the data required to replicate all study findings reported in the article, as well as related metadata and methods (https://journals.plos.org/plosone/s/data-availability#loc-minimal-data-set-definition).

For example, authors should submit the following data:

- The values behind the means, standard deviations and other measures reported;

- The values used to build graphs;

- The points extracted from images for analysis.

Authors do not need to submit their entire data set if only a portion of the data was used in the reported study.

If your submission does not contain these data, please either upload them as Supporting Information files or deposit them to a stable, public repository and provide us with the relevant URLs, DOIs, or accession numbers. For a list of recommended repositories, please see https://journals.plos.org/plosone/s/recommended-repositories.

If there are ethical or legal restrictions on sharing a de-identified data set, please explain them in detail (e.g., data contain potentially sensitive information, data are owned by a third-party organization, etc.) and who has imposed them (e.g., an ethics committee). Please also provide contact information for a data access committee, ethics committee, or other institutional body to which data requests may be sent. If data are owned by a third party, please indicate how others may request data access.

6. If the reviewer comments include a recommendation to cite specific previously published works, please review and evaluate these publications to determine whether they are relevant and should be cited. There is no requirement to cite these works unless the editor has indicated otherwise.

[Note: HTML markup is below. Please do not edit.]

Reviewers' comments:

Reviewer's Responses to Questions

**Comments to the Author**

1. Is the manuscript technically sound, and do the data support the conclusions?

Reviewer #1: Partly

Reviewer #2: Yes

Reviewer #3: Partly

Reviewer #4: Yes

**********

2. Has the statistical analysis been performed appropriately and rigorously?

Reviewer #1: Yes

Reviewer #2: No

Reviewer #3: N/A

Reviewer #4: Yes

**********

3. Have the authors made all data underlying the findings in their manuscript fully available?

The PLOS Data policy

Reviewer #1: Yes

Reviewer #2: Yes

Reviewer #3: Yes

Reviewer #4: Yes

**********

4. Is the manuscript presented in an intelligible fashion and written in standard English?

Reviewer #1: Yes

Reviewer #2: No

Reviewer #3: Yes

Reviewer #4: No

**********

Reviewer #1: In this manuscript titled "Identification of Flavonoids from Natural products against Hyperlipidaemia through

Computational Approaches", authors have evaluated pharmacological potential of flavonoids to identify strong inhibitors for target receptor, Proprotein Convertase Subtilisin/Kexin Type (PCSK9). The results revealed that the flavonoids evaluated, Tilianin, Myricetin, and Sinensetin demonstrate the most favourable interaction profiles.

These finding suggest that the selected flavonoids have strong potential as promising anti-hyperlipidaemic candidates targeting PCSK9.

Although the work seems interesting, a thorough revision is required before its consideration.

1) Abstract seems good with brief summary of the performed work.

2) Introduction: Seems weak with lack of research gaps. Authors are strongly encouraged to go through the following relevant research articles and include them accordingly.

https://doi.org/10.1016/j.ejphar.2020.173690

https://doi.org/10.1016/j.phymed.2023.155258

https://doi.org/10.1016/j.intimp.2024.112178

10.1038/s41401-024-01305-9

10.1021/acs.analchem.3c04746

https://doi.org/10.1016/j.molstruc.2024.139269

https://doi.org/10.1016/j.heliyon.2024.e37811

10.1039/D2FO02298B

https://doi.org/10.26599/FSHW.2024.9250007

3) Authors should provide more information on section 2.2.2 Refinement of PCSK9.

4) Can the authors detail on the chemical bonding involved in Structural Flexibility?

5) Conclusion section can involve numerical results (like abstract).

Reviewer #2: 1. The manuscript requires a thorough linguistic overhaul. There are numerous grammatical errors, typos, and poor phrasing choices throughout the mansucript that obscure the scientific content.

2. There is a noticeable gap in the biological rationale connecting the chosen plant extracts with the molecular targets. While targeting the PCSK9 pathway is a sound approach, the introduction fails to establish a clear, mechanistic link between flavonoids and this specific protein.

3. A critical accuracy issue arises regarding Talinum triangulare: the text claims its flavonoid extract (TTFE) addresses dyslipidemia by "enhancing insulin secretion through antioxidant mechanisms." However, established pharmacological literature (e.g., Oluba et al.) shows that TTFE’s lipid-lowering effects are primarily driven by the direct inhibition of the HMG-CoA reductase enzyme. The authors must correct this claim and provide the accurate bibliographical reference.

4. Furthermore, the manuscript needs to explain why flavonoids are viable candidates for this study. The authors should explicitly note that various flavonoid families are well-documented modulators of REBP-2 and HNF-1A, the key upstream transcription factors that dictate PCSK9 gene expression. This addition is vital to justify why these natural compounds are being screened against this specific target.

5. The Materials and Methods section currently lacks the depth required for reproducibility. To ensure the robustness of the in silico workflow, several technical protocols must be clarified.

First, the exact procedure for preparing and cleaning the initial coordinates from the Protein Data Bank (PDB) must be stated. It is necessary to clarify whether crystallographic water molecules, non-essential ions, co-crystals, or endogenous ligands were stripped from the structure, and whether missing loops or residues were reconstructed using homology modeling or specialized software packages.

Second, the system relaxation phase requires a more rigorous description. The manuscript should report the precise simulation times allocated for both the thermal equilibration under the canonical ensemble (NVT) and the pressure equilibration under the isothermal-isobaric ensemble (NPT). The specific thermostat and barostat algorithms used during these stages must also be named.

6. Finally, while the binding free energy calculations are noted as being performed via MM-PBSA over the stable 70–100 ns trajectory window, essential parameters are missing. The authors must specify the exact number of snapshots extracted for this analysis, disclose the precise values assigned to the solute and solvent dielectric constants, and break down the specific energetic contributions (such as van der Waals, electrostatic, polar, and non-polar solvation terms) included in the final estimations.

Reviewer #3: Reviewer comments:

Give a list of abbreviations for all abreviated terms found in the manuscript.

Revise the total binding free energy (MMPBSA) regarding some flavonoids with PCSK9, like sinensetin (−23.48 kcal/mol) and Eupatorin (−20.97 kcal/mol) that must align with the corresponding values in Fig.7.

Table 2:

What docking reference is used in this table, and how does the binding affinity score (kcal/mol) for each flavonoid compare to it?

Mention the RMSD of the docking reference to validate your docking process.

Provide further information regarding eupatorin's primary pharmacological actions in the introduction, along with any prior research on the drug's effects on cardiovascular disease or other pharmacological functions, all of which should be supported by references that support your hypothesis.

According to the author, Eupatorin essentially interacts with the catalytic domain of PCSK9 through allosteric modulation, inhibiting PCSK9 without directly preventing LDLR binding. Provide a more detailed explanation of allosteric modulation.

What is the evidence for this allosteric modulatory effect? A supporting figure or graph illustrating this type of interaction would be useful.

Find the primary structural characteristics of Eupatorin that support its allosteric modulatory activity.

.........................................................END............................................................

Reviewer #4: Overall revision command:

The manuscript presents an interesting computational screening of flavonoids

against PCSK9, but it requires substantial improvement in scientific rigor,

consistency, and presentation before it can be considered suitable for publication.

The intorduction of the article is not up-to-date, author requests will add the advanced and computational literature in introduction section. For in sililico and computational updapation, authors can follow the following articles:

1) https://doi.org/10.1016/j.molstruc.2021.131915

2) https://link.springer.com/article/10.1186/s43088-021-00117-8

3) https://doi.org/10.33945/SAMI/AJCA.2019.4.5

4) http://doi.org/10.25135/acg.oc.122.2203.2397

5) https://doi.org/10.33945/SAMI/AJCA.2019.2.190202

6) http://dx.doi.org/10.4067/S0717-97072021000205206

7) https://doi.org/10.1016/j.heliyon.2021.e07509

8) https://doi.org/10.3390/molecules27123694

9) https://doi.org/10.1016/j.molstruc.2021.131087

10) https://dergipark.org.tr/en/pub/tcandtc/article/481878

11) http://dx.doi.org/10.4067/S0717-97072022000305623

� Clarify the study design and workflow, because the manuscript alternates

between 28, 29, and 24 compounds and this inconsistency must be corrected

throughout the text, figures, and tables. Provide the flavonoid numbering in

Table 1.

� Recheck all numerical results and ensure they are internally consistent,

especially docking scores, MD descriptors, and MM-PBSA values, since

several values and ligand rankings appear to be presented differently in

different sections.

� Improve the methodological description so the refinement, docking, and MD

simulation steps can be reproduced precisely, including protein preparation,

ligand preparation, grid settings, simulation conditions, replicates, and

analysis parameters.

� Strengthen the rationale for choosing PDB ID 5OCA and explain more clearly

why this structure is appropriate for PCSK9-targeted inhibitor screening.

� Provide a more critical interpretation of the computational findings, because

favorable docking or MM-PBSA values alone are not sufficient to claim

therapeutic promise without experimental validation.

� Check reference formatting carefully, since several citations appear

incomplete or inconsistently styled.

Rewrite the conclusions regarding the objectives of introduction.

**********

what does this mean?). If published, this will include your full peer review and any attached files.

If you choose “no”, your identity will remain anonymous but your review may still be made public.

**Do you want your identity to be public for this peer review?**  For information about this choice, including consent withdrawal, please see our Privacy Policy

Reviewer #1: No

Reviewer #2: No

Reviewer #3: No

Reviewer #4: No

**********

[NOTE: If reviewer comments were submitted as an attachment file, they will be attached to this email and accessible via the submission site. Please log into your account, locate the manuscript record, and check for the action link "View Attachments". If this link does not appear, there are no attachment files.]

To ensure your figures meet our technical requirements, please review our figure guidelines: https://journals.plos.org/plosone/s/figures

You may also use PLOS’s free figure tool, NAAS, to help you prepare publication quality figures: https://journals.plos.org/plosone/s/figures#loc-tools-for-figure-preparation.

NAAS will assess whether your figures meet our technical requirements by comparing each figure against our figure specifications.

# Author comment: Author response to Decision Letter 1

**DOI:** 10.1371/journal.pone.0346073.r002

19 Aug 2026

Dear Academic Editor and Reviewers,

We sincerely thank the Academic Editor and all reviewers for their careful evaluation of our manuscript and for providing constructive and valuable comments. We have carefully considered all comments and have revised the manuscript accordingly. The revisions have improved the clarity, methodological reproducibility, scientific rationale, consistency of the results, and interpretation of the computational findings.

All changes have been incorporated into the revised manuscript, and the relevant sections, tables, figures, and supplementary materials have been updated where appropriate. Our point-by-point responses are provided in the document with file name "Response to Reviewer".

We sincerely thank the Academic Editor and all reviewers for their constructive comments. Their suggestions have helped us improve the scientific clarity, methodological transparency, consistency, and interpretation of the manuscript. We believe that the revised manuscript addresses the concerns raised and provides a more rigorous and balanced presentation of the potential of flavonoids as PCSK9-targeting compounds.

We respectfully submit the revised manuscript for further consideration by PLOS ONE.

Thank you

Attachment Submitted filename: Response to Reviewers.docx

# Peer review: Decision Letter 1

**DOI:** 10.1371/journal.pone.0346073.r003

**Authors:** Jorddy Neves Cruz

17 Sep 2026

Identification of Flavonoids from Natural products against Hyperlipidaemia through Computational Approaches

PONE-D-26-12566R1

Dear Dr. Ismail,

We’re pleased to inform you that your manuscript has been judged scientifically suitable for publication and will be formally accepted for publication once it meets all outstanding technical requirements.

Within one week, you’ll receive an e-mail detailing the required amendments. When these have been addressed, you’ll receive a formal acceptance letter and your manuscript will be scheduled for publication.

An invoice will be generated when your article is formally accepted. Please note, if your institution has a publishing partnership with PLOS and your article meets the relevant criteria, all or part of your publication costs will be covered. Please make sure your user information is up-to-date by logging into Editorial Manager at Editorial Manager® and clicking the ‘Update My Information' link at the top of the page. For questions related to billing, please contact billing support.

If your institution or institutions have a press office, please notify them about your upcoming paper to help maximize its impact. If they’ll be preparing press materials, please inform our press team as soon as possible -- no later than 48 hours after receiving the formal acceptance. Your manuscript will remain under strict press embargo until 2 pm Eastern Time on the date of publication. For more information, please contact onepress@plos.org.

Kind regards,

Jorddy Neves Cruz

Academic Editor

PLOS One

Additional Editor Comments (optional):

Reviewers' comments:

Reviewer's Responses to Questions

**Comments to the Author**

Reviewer #1: All comments have been addressed

Reviewer #2: All comments have been addressed

**********

2. Is the manuscript technically sound, and do the data support the conclusions?

Reviewer #1: Yes

Reviewer #2: Yes

**********

3. Has the statistical analysis been performed appropriately and rigorously?

Reviewer #1: N/A

Reviewer #2: Yes

**********

4. Have the authors made all data underlying the findings in their manuscript fully available?

The PLOS Data policy

Reviewer #1: Yes

Reviewer #2: Yes

**********

5. Is the manuscript presented in an intelligible fashion and written in standard English?

Reviewer #1: Yes

Reviewer #2: Yes

**********

Reviewer #1: Authors have addressed all the given comments satisfactorily. This can be considered for publication.

Reviewer #2: The authors have satisfactorily addressed all comments from the initial review, clarifying the methodological details of the molecular dynamics and MM/PBSA calculations. The methods section is now clear, rigorous, and transparent, ensuring the reproducibility of the study.

**********

what does this mean?). If published, this will include your full peer review and any attached files.

If you choose “no”, your identity will remain anonymous but your review may still be made public.

**Do you want your identity to be public for this peer review?**  For information about this choice, including consent withdrawal, please see our Privacy Policy

Reviewer #1: No

Reviewer #2: No

**********

# Editor report: Acceptance letter

**DOI:** 10.1371/journal.pone.0346073.r004

**Authors:** Jorddy Neves Cruz

PONE-D-26-12566R1

PLOS One

Dear Dr. Ismail,

I'm pleased to inform you that your manuscript has been deemed suitable for publication in PLOS One. Congratulations! Your manuscript is now being handed over to our production team.

At this stage, our production department will prepare your paper for publication. This includes ensuring the following:

* All references, tables, and figures are properly cited

* All relevant supporting information is included in the manuscript submission,

* There are no issues that prevent the paper from being properly typeset

You will receive further instructions from the production team, including instructions on how to review your proof when it is ready. Please keep in mind that we are working through a large volume of accepted articles, so please give us a few days to review your paper and let you know the next and final steps.

Lastly, if your institution or institutions have a press office, please let them know about your upcoming paper now to help maximize its impact. If they'll be preparing press materials, please inform our press team within the next 48 hours. Your manuscript will remain under strict press embargo until 2 pm Eastern Time on the date of publication. For more information, please contact onepress@plos.org.

You will receive an invoice from PLOS for your publication fee after your manuscript has reached the completed accept phase. If you receive an email requesting payment before acceptance or for any other service, this may be a phishing scheme. Learn how to identify phishing emails and protect your accounts at https://explore.plos.org/phishing.

If we can help with anything else, please email us at customercare@plos.org.

Thank you for submitting your work to PLOS One and supporting open access.

Kind regards,

PLOS One Editorial Office Staff

on behalf of

Dr. Jorddy Neves Cruz

Academic Editor

PLOS One
